# Using eDNA sampling for species-specific fish detection in tropical oceanic samples: limitations and recommendations for future use

**DOI:** 10.7717/peerj.14810

**Published:** 2023-02-02

**Authors:** Giovanna M. Gonzalez Colmenares, Alejandro J. Gonzalez Montes, Chelsea A. Harms-Tuohy, Nikolaos V. Schizas

**Affiliations:** 1 Department of Biology, Universidad de Puerto Rico, Recinto de Mayagüez, Mayagüez, Puerto Rico; 2 Department of Marine Sciences, Universidad de Puerto Rico, Recinto de Mayagüez, Mayagüez, Puerto Rico; 3 Isla Mar Research Expeditions, Rincón, Puerto Rico

**Keywords:** eDNA sampling, Species-specific detection, Fish spawning aggregations, Tropical eDNA, Fisheries management

## Abstract

**Background:**

Over the past decade, environmental DNA (eDNA) has become a resourceful tool in conservation and biomonitoring. Environmental DNA has been applied in a variety of environments, but the application to studies of marine fish, particularly at tropical latitudes, are limited. Since many commercially important Caribbean fishes are overexploited, these species are optimal candidates to explore the use of this method as a biomonitoring tool. Specifically, for many of these species, the formation of fish spawning aggregations (FSAs) marks a critical life history event where fishes will gather in large numbers for reproduction. These FSAs are ephemeral in nature, lasting only a few days, but are predictable in time and space which makes them susceptible to overfishing.

**Methods:**

In this study, we test the feasibility of using an eDNA sampling approach (water and sediment collection) to detect the presence of known FSAs off the west coast of Puerto Rico, with cytochrome c oxidase subunit 1 (CO1) and 12S rRNA (12S) primers designed to target specific species. A total of 290 eDNA samples were collected and, of those, 206 eDNA samples were processed. All eDNA samples varied in DNA concentration, both between replicates and collection methods. A total of 12 primer sets were developed and tested using traditional PCR and qPCR.

**Results:**

Despite validation of primer accuracy and sample collection during known peak spawning times, the use of traditional PCR and qPCR with both molecular markers failed to produce species-specific amplification. Thus, a trial test was conducted using the CO1 primers in which target fish DNA was ‘spiked’ at various concentrations into the respective eDNA samples to determine the target species DNA concentration limit of detection. Upon successful amplification of the trial, results indicated that eDNA samples were below the detection threshold of our methods, suggesting that the number of fish present at the spawning aggregations was inadequate for single-species detection methods. In addition, elements such as the unavoidable presence of non-target DNA, oceanic environmental conditions, shedding rates of target fish, among other biotic and abiotic factors could have affected DNA persistence and degradation rates at the sites.

**Conclusion:**

We provide recommendations for species-specific fish detection in lower latitudes, and suggestions for studies aiming to monitor or detect fish spawning aggregations using eDNA sampling.

## Introduction

Many commercially important fish species aggregate at specific times and places for the purpose of reproduction. The number of individuals present at the timing of aggregation formation is significantly higher (100s–1,000’s depending on the species) than those abundances found during the non-reproductive periods (Nemeth, 2009; Sadovy de Mitcheson & Colin, 2011). The occurrence of these events depends on the availability of suitable areas for feeding and breeding (Claydon, 2004), as well as other factors such as the lunar cycles, diurnal variations, and temperature and current alterations (Sadovy de Mitcheson, 1996; Samoilys, 1997; Nemeth, 2009). Species such as groupers and snappers are commonly known to travel great distances to these aggregation sites, which can be shared by more than one species and sometimes even overlap in timing and duration (Heyman & Kjerfve, 2008). The predictability of these natural events makes them an easy target for fisheries and overexploitation (Sadovy de Mitcheson, 2016; Sadovy & Domeier, 2005). The Caribbean is an unfortunate example highlighting the exploitation of fish spawning aggregations. Some of these species, such as the Nassau grouper (*Epinephelus striatus*), have been listed as critically endangered by the International Union for Conservation of Nature (IUCN)(Sadovy de Mitcheson, Aguilar-Perera & Sosa-Cordero, 2018). Nassau grouper spawning aggregations have declined by 60% in the Caribbean (Sadovy de Mitcheson et al., 2008), and “fishing down the food web” has led to the decline of other groupers, such as red hind (Sadovy de Mitcheson, 1999; Beets & Friedlander, 1992) along with commercially important species like mutton snapper (*Lutjanus analis*) and yellowfin grouper (*Mycteroperca venenosa*) (Luckhurst, 2003; Nemeth et al., 2006). Many exploited fish families such as Haemulidae (grunts), Lutjanidae (snappers), and Serranidae (groupers) have been associated with maintaining a healthy reef ecosystem (Grober-Dunsmore et al., 2007). Considering their ecological and economical importance, the protection and conservation of these aggregation-forming fish species are imperative for the overall resilience of marine food webs.

Fish spawning aggregations (FSAs) have been documented and monitored using multiple field- and personnel-intensive approaches. Some of these methods include fish counting *via* underwater visual census, which oftentimes is impaired by the sheer number of individuals present. Tens of thousands of fish in constant movement can be influenced by currents and the presence of divers, which can also influence how fish interact and behave during spawning (Kobara & Heyman, 2008). Other methods to first locate FSAs include hydroacoustic surveys such as echosounders (Binder et al., 2021) and underwater microphones (hydrophones) (Appeldoorn et al., 2013; Chérubin et al., 2020). Some of these methods can also be used to monitor FSAs (Schärer et al., 2012), among other methods such as digital imaging using baited or non-baited video cameras (Cappo, Harvey & Shortis, 2006), acoustic tagging (Tuohy et al., 2015), and remote sensing (Kobara & Heyman, 2007). However, all these methods can also be affected by visibility and ocean conditions, and can be costly or require skilled personnel that may not always be available year after year for aggregation monitoring. As a result, obtaining accurate and precise data is not only difficult but can also be costly (Pfrender et al., 2010), and these are just some of the challenges to monitoring known aggregations that are accessible to relocate annually.

Although these methods have been successfully used to monitor FSAs, there remains a need to find a method that detects spawning aggregations in a cost-effective, rapid manner. One promising approach is using environmental DNA (eDNA). Ikeda et al. (2019) proposed eDNA as an efficient and highly sensitive method to assess the distribution of aquatic organisms. The eDNA sampling approach has also been suggested to have the potential to revolutionize marine biomonitoring (Andruszkiewicz, Sassoubre & Boehm, 2017). Environmental DNA is a relatively young field in molecular ecology which uses DNA obtained from the environment (Bohmann et al., 2014) with the goal of identifying a specific organism or group of organisms present in a certain time and space without the need to isolate or capture the organism (Stewart, 2019; Wang et al., 2021). The eDNA sampling technique has also been used successfully to locate and identify a variety of freshwater and temperate latitude marine macro-organisms including those associated with fish spawning aggregations. For example, Ratcliffe et al. (2021), focusing on egg and larval stages, used eDNA sampling while simultaneously collecting ichthyoplankton to compare taxa detection for monitoring purposes. Tsuji & Shibata (2021) identified fish spawning events by detecting a spike in eDNA concentration in a laboratory setting. In field studies, both Tillotson et al. (2018), and Hayer et al. (2020) used eDNA sampling to detect spawning events in constrained freshwater locations. The use of eDNA sampling to detect FSAs in open marine environments has not been studied, but these studies suggest eDNA sampling has the potential to detect and assess FSAs of threatened and commercially important species.

The residency of eDNA in biological systems is influenced specifically by four factors: origin, state, transport, and fate of the DNA (Turner, Uy & Everhart, 2015). In aquatic environments, feces have been identified as one of the major sources of fish DNA, but these can easily become diluted in water, so samples must be collected as close to the source as possible (Caldwell, Payment & Villemur, 2011). Specifically for marine systems, the transport of DNA could refer to the movement through the water column, currents, or settling down in the benthos. Biotic factors such as the density of the organism or group of organisms present in the environment (Dejean et al., 2011), the organism’s size and life history stage, immunological state, reproductive status, metabolic rate, or stress to which the organism is being subjected (Sassoubre et al., 2016; Stewart, 2019) influence the condition of DNA in the system. Feeding, diet, and migration also influence DNA concentration, as does the shedding rate (Rourke et al., 2022). Abiotic factors such as salinity, pH, temperature, UV radiation, water currents and dilution processes in the water column (Sassoubre et al., 2016; Ficetola et al., 2008) can influence the residency and decay of DNA (Stewart, 2019). Additionally, water current speed and direction, as well as tidal fluctuations, can influence the direction and transport DNA downstream from the source, which could result in false-positive detections (Jeunen et al., 2019). For this reason, it is imperative to understand the dynamics of the study system in question and consider all the possible factors of the environment in which the samples will be collected in order to facilitate the greatest chance for successful DNA detection.

Choosing the right genetic marker can be difficult, specifically for eDNA studies which require smaller barcodes since DNA is mostly found fragmented or degraded when sampled directly from the environment. Thus, using or designing primers should be limited to roughly 200–250 bp to increase PCR success rate (Freeland, 2017; Bylemans et al., 2018; Zhang, Zhao & Yao, 2020). Mitochondrial genes are often used in eDNA metabarcoding (or, in general, DNA barcoding) studies because of their high efficiency for detection among degraded DNA and their availability in reference databases. These genes have been used to assess the biodiversity of fish communities (Shu, Ludwig & Peng, 2020), the phylogenetic diversity of bony fish and elasmobranchs (Marques et al., 2021), and fish population connectivity (Okumuş & Çiftci, 2003). Two of the most popular genetic markers for these studies include Cytochrome c Oxidase Subunit 1 (CO1) and 12S rRNA (12S). The amplified fraction of the CO1 gene is usually about 650 base pairs, depending on the primers, and is highly efficient as a genetic marker commonly used for universal DNA barcoding for metazoans (Rach et al., 2017). The CO1 gene has been previously used for the detection and identification of aquatic macro-organisms (Zemlak et al., 2009; James, Bolick & Suzumoto, 2010; Deiner et al., 2013). The 12S marker, a portion of the small mitochondrial rRNA gene, is commonly used to detect a wide variety of aquatic organisms, including fish (Polanco et al., 2021; Gold et al., 2021). Both markers have been previously used in eDNA biodiversity studies to estimate species richness (Shu, Ludwig & Peng, 2020), compare, and detect fish communities (Hallam et al., 2021) and for monitoring fish communities in spawning areas (Ratcliffe et al., 2021). The CO1 marker has also been used for detecting invasive marine species (LeBlanc et al., 2020), for species-specific studies (Wang et al., 2020) and for detecting spawning distributions of European anadromous shads (Antognazza et al., 2019) but there is no evidence to support CO1 or 12S as the most effective marker for species-specific detection in tropical marine environmental samples.

In this study, we apply the use of eDNA sampling to the tropical Caribbean waters of Puerto Rico. The focus of this study was to test the effectiveness of three different eDNA collection methods and targeted detection of specific fish species using two molecular markers (CO1 and 12S). In addition, a comparison of molecular techniques between traditional polymerase chain reaction (PCR) and quantitative PCR (qPCR) was performed with both genes. Our target organisms were four commercially important fishery species that form well-documented FSAs including red hind (*Epinephelus guttatus*), Nassau grouper (*Epinephelus striatus*), yellowfin grouper (*Mycteroperca venenosa*) and mutton snapper (*Lutjanus analis*). These species were chosen because of their commercial importance in Puerto Rico and based on long-term monitoring of their spawning aggregations, where abundance estimates are well known during the time of aggregation formation. The results highlight the challenges of working with eDNA in open ocean tropical latitudes and provide species-specific primer sets for future use in eDNA studies.

## Materials & Methods

### Equipment Preparation

Before each sampling event, all field and laboratory equipment were sterilized, labeled, and prepared one day before sampling to reduce contamination risk. Water samples were collected in individual two-liter silicone, pressure-resistant bottles with screw-top lids (Hydrapak Seeker™ 2L, Hydrapak, Oakland, CA, USA). Sediment samples were collected in individual 50 ml plastic centrifuge tubes (VWR™, Radnor, PA, USA). Prior to sampling, each bottle and tube received an extensive sterilization procedure. The Hydrapak bottles were washed with freshwater and submerged in a 20% bleach solution for 30 min. Subsequently, the bottles were rinsed in freshwater (∼8–10 washes), cleaned with 99% ethanol, and then left open to air dry in a sterile space. Once dried, the bottles underwent UV light exposure for 15 min. Centrifuge tubes were autoclaved, placed under UV light for 15 min and filled with distilled water. Distilled water was used to ensure the tubes did not collapse under pressure when taken to depth to collect the sediment samples. All sampling equipment was stored in triplicates in their own sterilized plastic bag which was labeled based on the sampling method used. Bottles were labeled according to manner of sampling. Control samples of distilled water were also prepared prior to each sampling event using Hydrapak bottles that underwent the same transportation as the samples collected at the sites. The filtering station was cleaned using bleach wipes and ethanol before each sample processing event and in between filtering different samples. Sterile, individually packaged single-use filters were placed under UV light for 15 min before use.

### Sample collection sites

**Figure 1:**
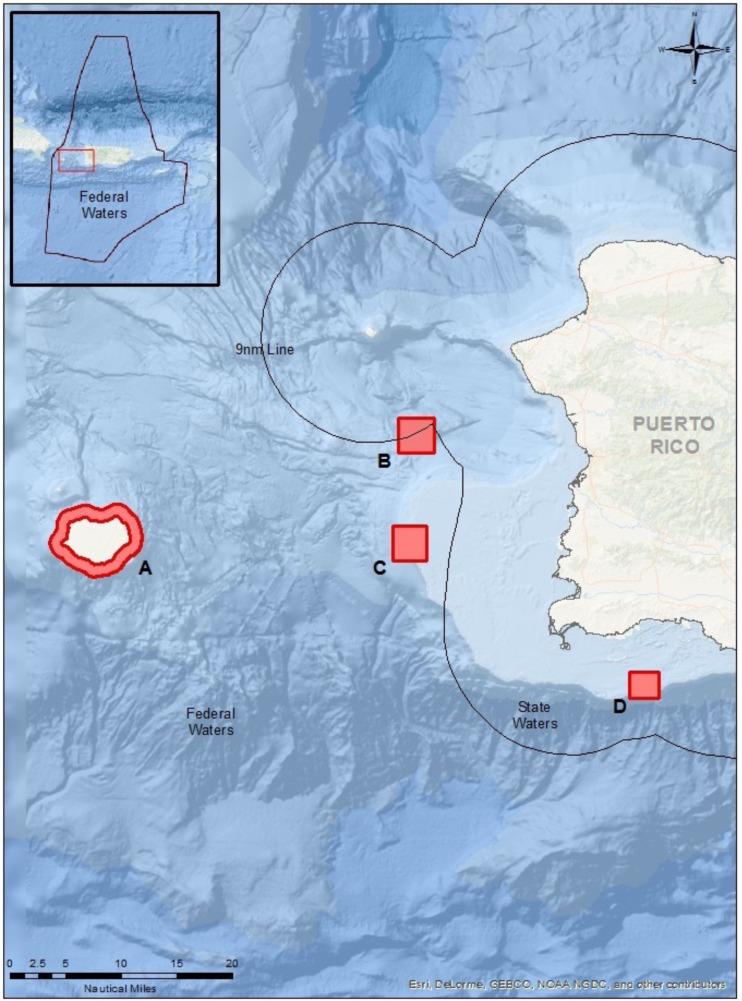
Map of sampling locations in Puerto Rico (see lines of 9 nautical miles and 200 nautical miles in insert) for the known FSA sites. (A) Mona Island, (B) Bajo de Sico, (C) Abrir la Sierra Bank, and (D) La Parguera.

The eDNA samples were collected off the west coast of Puerto Rico for the four target species at their respective known spawning sites (Fig. 1) during the aggregation periods from January to May 2019 (Table 1). Sampling for each species aggregation was targeted during the species’ most active (highest fish count) aggregation month corresponding to the lunar cycle. For example, a species that forms an FSA during January to March, with January being the historically highest fish abundance, was only sampled during January. The chosen month was based on long-term monitoring of these FSA sites (Sadovy de Mitcheson, 1999; Ojeda-Serrano, Appeldoorn & Ruź-Valentín, 2007; Schärer, Nemeth & Appeldoorn, 2010; Kobara et al., 2013). In some cases, due to weather conditions, additional sampling may have occurred during the species’ other aggregation months and some sampling methods could not be conducted at the FSA sites due to logistical constraints of sampling at deep depths. All sample collection, equipment deployment, and monitoring were conducted under a local permit (DRNA scientific permit #2018-IC-075).

**Table 1:** The number of eDNA samples collected per sampling method and per fish species. The sampling month denotes that only one target fish species was sampled at each eDNA sampling event during that time frame, corresponding to the FSA peak. N Fish is maximum count of that species observed by divers on the sampling day.

Target species	Year	Month	Diver	VanDoorn	Sediment	Control	Total	N Fish
Red hind	2019	Late January	24	12	30	30	96	32
Nassau grouper		Early March	9	6	12	24	51	58
Yellowfin grouper		Late March–April	20	12	27	30	89	45
Mutton snapper		Late April–May	21	9	24	24	78	0

### Sampling methods

Three different eDNA collection methods were used to obtain water and sediment samples at these FSA sites: (1) Diver (D, *n* = 3 per sampling event), in which the water sample was collected at depth at the exact location of aggregation formation, (2) VanDoorn (V, *n* = 3), in which a horizontal point water sampler was lowered from the boat to the depth of the relative location of main aggregation formation, and (3) Sediment (S, *n* = 3), where sand and sediment were collected at the main aggregation site by divers. More specifically, the D method consisted of collecting water from the water column by divers using the Hydrapak bottles. The V method consisted of collecting water with a 2.2 L polycarbonate Horizontal Point Water Sampler (Aquatic Research, Hope, ID, USA) which was lowered from the boat to the aggregation depth. Once the bottle reached the depth, a messenger weight was sent down the line to close the bottle before hauling it back to the surface. Water collected using the V method was transferred to labeled Hydrapak bottles for storage on board the vessel. The VanDoorn sampler was cleaned in between sampling using 15% bleach, rinsed and then followed by 95% ethanol to ensure sterilization between samples. The S method consisted of divers collecting a full tube of sediment underneath the aggregation using the triplicate 50 mL tubes. In addition to the distilled water controls, ocean surface water was collected at the aggregation site as an additional control since target DNA was not expected to persist at the surface based on the aforementioned factors of transport and degradation. For each sampling event, a total of 12 water samples (D, V and two controls) and three sediment samples were collected. However, in some cases, the total sample count differed slightly from this intended plan due to conditions underwater at the time of sampling that prevented collection (see Table 1). These volumes were chosen based on several factors: (1) the available published methodologies at the time of the study’s initiation, (2) the volumes which our team could efficiently process in a timely manner on the same day as collection in the laboratory setup and (3) the volumes at which the dive team could quickly and effectively collect given the often-variable oceanic conditions during FSA formation months. Given these conditions, additional consideration was given to the time of sampling (*i.e.,* sampling on peak day) in order to ensure that the largest amount of target DNA was present. To ensure sampling on the peak day, divers performed underwater visual census on days leading up to the expected peak and on days following the peak to estimate fish abundance.

In addition, a non-aggregation site was also sampled in the same way (*n* = 3 water samples, 3 sediments, 2 controls) on the same peak day to serve as a site control (Table 1). Thus, in total, there were 113 water samples (D, V), 93 sediment samples and 84 control samples (both water and sediment) for an overall total of 290 eDNA samples that were collected for this project. All samples were immediately placed on ice to ensure preservation. Samples were transported to the University of Puerto Rico’s, Isla Magueyes Marine Genomics Laboratory and remained on ice prior to filtration.

**Table 2:** The CO1 and 12S primers used and designed for the target species of interest.

Target species	Gene	Primer name	Tm (°C)	Sequence 5′–3′	Reference
Metazoan diversity	CO1	F: mlCOIintF, R: jgHCO2198	51.2 46.4	GGWACWGGWTGAACWGTWTAYCCYCC TAIACYTCIGGRTGICCRAARAAYCA	Leray et al. (2013)
Red hind	CO1	Epine_gutt_For, Epine_gutt_Rev	57.6 57.4	ACCCGCATGGGCTAGATTTC TTCGTCTGGAGTTGAAGCCG	Made with Primer Blast
Nassau grouper	CO1	Epi_str_For, Epi_str_Rev	58.4 56.4	GCTGGACGGTATATCCCCCT GATACTGGGAGATTGCGGGA	Made with Primer Blast
Mutton snapper	CO1	Lut_ana_For, Lut_ana_Rev	56.4 58.5	CGCTATTCGTTTGAGCCGTC AGATGGCAGGGGGCTTCATA	Made with Primer Blast
Subtropical marine fishes	12S	MiFish-U Forward MiFish-U Reverse	56.6 56.5	GTCGGTAAAACTCGTGCCAGC GTATCACCCCATAGATTAGGGTCAAAC	Miya et al. (2015)
Red hind	12S	RH-F 12S, RH-R 12S	53.8 52.7	GGAACGCTCTGCTTTCTG GGCTACATTCCCTGTCAC	GenBank: AY949437
Nassau grouper	12S	NS-F 12S, NS-R 12S	57.6 56.6	TCTAAAGCACCGCCAAGTCC ACACAATAACTATCCGCCTGGAG	GenBank: AY949433
Yellowfin grouper	12S	YF-F 12S, YF-R 12S	51.2 53.2	GTAATAGGGAATGTAGCCCA GGCCCTTCAGTAAGCAC	GenBank: AY949419
Mutton snapper	12S	MT-F 12S, MT-R 12S	56.3 56.2	CGCCTATATACCACCGTCGT TCCATACGCTACACCTCGAC	GenBank: EF095569

### Tissue collection

Tissue samples were collected from the target species using TissueGrab™ biopsy tips (DRNA scientific permit #2018-IC-075; Pelagic Research Group, LLC, Honolulu, HI, USA; DRNA scientific permit #2018-IC-075) to assist in preparation of the species-specific primer sets. The biopsy tips do not penetrate the flesh of the skin but rather briefly stick and bounce off with a small amount of tissue or scales collected in the tip point. This collection method was a necessary way to ensure exact genetic material was obtained to represent our local aggregations, but also prevented us from killing these species for this purpose. Tissue samples were placed in 2.5 mL 95% ethanol-filled tubes and stored at −20 °C until extraction.

### Biological and physical data collection

In addition to eDNA sampling, a visual census to acquire fish count and biomass was also recorded on each sampling day. The purpose of the fish survey was to estimate the number of fish present during each eDNA sampling event to relate the number of individuals to the amount of DNA obtained from the samples. The fish counts also allowed us to validate that we sampled during the peak times of aggregation formation. Additionally, digital spectrogram recorders (DSGs) were deployed at each aggregation, allowing us to listen to courtship associated sounds (CAS) and validate the presence of each target species at the aggregation (Schärer et al., 2012), which further supported the timing of our sampling.

To obtain a more robust understanding of oceanic conditions at the exact time of sampling, we deployed an Acoustic Wave and Current Meter (AWAC) (Nortek, Providence, RI, USA) at three of the four aggregation sites prior to sampling days. This allowed us to determine the speed, direction, and strength of the current at depth and through the water column to the ocean surface for each of our sampling days. An analysis of the hydrodynamic conditions (current velocity, direction at the time of sampling) was created for each aggregation site.

### Sample processing

#### Filtration and DNA extraction

Samples were filtered in a designated fume hood used only for this project, where two samples could be filtered simultaneously using two peristaltic pumps. Each water sample was processed through a pre-packaged, sterilized, single-use 47 mm diameter cellulose nitrate filter with a 0.22 µm pore size (Nalgene, Rochester, NY, USA) (Hinlo et al., 2017; Bracken et al., 2019; Djurhuus et al., 2018; Schabacker et al., 2020). Filtering time per sample was roughly 30 to 35 min with an average of seven hours required to filter one sampling event. After filtering, each filter was placed in a labeled and sterilized plastic bag using sterile tweezers and stored at −20 °C until DNA extraction. Sediment samples were not filtered but were instead separated into two 50 ml centrifuge tubes and filled with 95% ethanol for preservation and stored at −20 °C until downstream processing.

DNA extractions were performed in a designated extraction site separate from the filtering station using a modified Qiagen DNeasy Blood and Tissue Extraction kit (Qiagen, Hilden, Germany) protocol. The Qiagen DNeasy kit is widely used in marine macro-organism eDNA studies (Thomsen et al., 2012; Andruszkiewicz, Sassoubre & Boehm, 2017; Takeuchi et al., 2019; Preißler et al., 2019; Shu, Ludwig & Peng, 2020; Rourke et al., 2022). Modifications consisted of thawing the filters for 3–5 min (Wilson et al., 2014), cutting the filter in half, and placing one of the halves in a 1.5 mL microcentrifuge tube (VWR) and storing at −20 °C for backup. The half used for the extraction was shredded using sterile scissors (Preißler et al., 2019) which were sterilized between samples and placed in 500 µL of ATL buffer and 40 µL of proteinase K in a 1.5 mL tube containing the filter for extraction, vortexed briefly and incubated at 56 °C for four hours (Preißler et al., 2019; K Deiner, 2019 pers. comm.). After incubation, the filter was carefully removed using sterilized tweezers and discarded. An additional 500 µL of ethanol and AL buffer were added to the tube and the remnants were vortexed before undergoing the remaining DNeasy Blood and Tissue Kit protocol guidelines.

Sediment samples (in pairs) were left to thaw at room temperature, homogenized by shaking and left to rest for five minutes. After homogenization, 500 µL from each sediment sample and its duplicate were pipetted into a 2.5 mL tube for a total of 1000 µL. Samples were then placed in the centrifuge 5810 R (Eppendorf, Hamburg, Germany) at 22,000g for 8 min at 4 °C. The supernatant was discarded and 180 µL of ATL buffer and 20 µL of proteinase K was added. Samples were briefly vortexed and incubated for four hours at 56 °C. After incubation, DNA was extracted from the sediment samples using DNeasy’s Blood and Tissue kit following manufacturer protocol. Finally, DNA from the tissue samples was extracted using DNeasy Blood and Tissue kit following the manufacturer’s protocol without any modifications. DNA concentrations were measured for each sample using a Nanodrop 2000/c spectrophotometer (Thermo Fisher Scientific, Waltham, MA, USA). All extractions were stored at −20 °C until further processing.

#### Primer development

CO1 species-specific primers were developed (Table 2) after amplifying tissue from each target species. Primers used to amplify the marker were mlCOIintF (5′-GGWACWGG WTGAACWGTWTAYCCYCC-3′) forward primer and jgHCO2198 (5′-TAIACYTCIGGRTGICCRAARAAYCA-3′) reverse primer to target a 313 bp region of the CO1 marker (Geller et al., 2013; Leray et al., 2013). The reaction was as follows: 10 µL KAPA Taq Ready Mix (Sigma-Aldrich, St. Louis, MO, USA), 3–5 µL from template tissue DNA, 1.0 µL of forward and reverse primer (CO1) and finally PCR grade water to complete a total of 20 µL reaction. PCR protocol was as follows: initial denaturing at 94 °C for 4 min, denaturing at 94 °C for 30 s, annealing 51 °C for 1 min, extension at 72 °C for 1 min, followed by 40 cycles and finished with a final extension of 72 °C for 1 min. After amplification, the PCR products were Sanger sequenced (MCLab, South San Francisco, CA, USA) and the resulting sequences were matched to the targeted species using BLAST (NCBI). After confirming identity, species-specific CO1 primers were designed using Primer BLAST. Protocol for the reaction was the same as stated above with changes in annealing temperature for each species (Table 2). Primers were tested within and between species. For each set of primers, PCRs were performed with the four targeted species’ DNA to confirm that each set only amplified the species for which it was designed. For example, a PCR was performed with red hind, Nassau, yellowfin, and mutton DNA using only the red hind primers. This procedure was conducted for all four species, changing the primer set until all four primers were tested for all species. PCR reaction and protocols were performed as follows: 10 µL of AmpliTaq Gold 360 master mix (Applied Biosystems, Waltham, MA, USA), with 0.8 µL of forward and reverse primers, 6.4 µL of PCR grade water and 2 µL of tissue DNA for a final reaction volume of 20 µL. For the PCR protocol, an initial denaturing at 95 °C for 5 min, denaturing at 95 °C for 30 s, annealing at 57.4 °C for 30 s, extension at 72 °C for 1 min repeated for 30 cycles and finished with a final extension time of 72 °C for 7 min and a final hold of 4 °C. The same reaction and protocol were performed with each set of primers for each species including all four targeted species in the same run. Primers and annealing temperatures were changed respectively. No primers were developed for yellowfin as DNA amplification was not successful from the small amount of tissue/scale that was retrieved from the biopsy tip.

The 12S species-specific primers were created using reference sequences retrieved from NCBI (Table 2) and using Primer BLAST for the four targeted species to ensure primer specificity. The 12S species-specific markers were designed as 100 bp barcode for each of the four species. Shorter markers (<200 bp) are best suited for qPCR because larger markers result in low efficiency and could account for errors and non-specific amplification while short primers provide greater efficiency for detecting most taxa at the species level (Vamos, Elbrecht & Leese, 2017).

#### eDNA sample amplification

To amplify the target DNA in the respective eDNA samples, a PCR reaction of 12.5 µL of AmpliTaq Gold 360 master mix (Applied Biosystems™), with 1.0 µL of forward and reverse primers specific to the target species, 6.4 µL of PCR grade water and 2 µL of specific species template DNA for a final reaction volume of 25 µL. PCR protocol was as follows: initial denaturing at 95 °C for 5 min, denaturing at 95 °C for 30 s, annealing at 57.4 °C for 30 s, extension at 72 °C for 1 min repeated for 30 cycles and finished with a final extension time of 72 °C for 7 min. The same protocol was conducted for all samples with the changes in annealing temperature based on the primer specifications. PCR products were visualized on 1.5% gel electrophoresis and placed under UV light to verify amplification, after which the PCR products were sequenced as a final validation step by a third-party lab (MCLAB, California).

Real Time PCR was conducted (CFX96 Dx Real-Time PCR Detection Systems; Bio-Rad) for each of the aggregations with sample triplicates. However, first to confirm the reliability of the results, several quality control steps were performed prior to processing any of the eDNA samples. First, a melt curve was prepared using temperature increments to determine the correct temperature for fluorescence per target species, given that the primer sets had different annealing temperatures. Standard curves were developed for each aggregation using tissue DNA (pure concentrated DNA) and a dilution series in concentrations of 1:10, 1:100 and 1:1,000 to determine the limit of detection. The real-time PCR was carried out using 10 µL of SYBR^®^ Green PCR Master Mix, 0.4 µL of species-specific primers, PCR water and 2.0−5.0 µL template DNA, following the manufacturer’s guide. The protocol was composed of initial denaturing at 95 °C for 3 min, denaturing at 95 °C for 10 s, annealing temperature depending on the primers using 35-55 cycles following a melt curve. Once these QAQC steps were performed, then eDNA samples were tested. A No Template control (NTC, negative control) was placed between the positive control (PC, tissue sample) and eDNA samples from the aggregations.

As validation for our in-house processing, selected samples from the red hind, Nassau, and yellowfin grouper eDNA samples were also sent out to a third-party lab (Laragen, Inc., Culver City, CA, USA). The third-party lab protocol using qPCR was done in 25 µL reactions composed of 12.5 µL of master mix, 0.5 µL of our species-specific forward and reverse primers, 9.5 µL of distilled H_2_O and 2 µL of DNA template.

Finally, a separate protocol was performed to identify the concentration of DNA required to detect the target species within the eDNA samples. The purpose was to identify if the concentrations of target fish DNA within our samples were potentially too low to be detected. To perform this test, Nassau grouper aggregation eDNA samples were spiked with various concentrations of Nassau tissue DNA (0.25 µL–1 µL). The protocol was performed as follows: initial denaturing 95 °C for 4 min, denaturing 94 °C for 30 s, annealing 56.4 °C for 45 s and extension at 72 °C for 30 s followed by 40 cycles and finalized with a final extension of 72 °C for 3 min. A 25 µL reaction was performed with 12.5 µL of KAPA Taq Ready Mix (Sigma-Aldrich™), 0.4 µL of forward and reverse primer (Table 2), 1.0 µL of BSA, 5.7 µL of PCR-grade water and 5.0 µL of template DNA, the same samples used were “spiked” with 1.0 µL of Nassau grouper DNA with a concentration of 79 ng/ µL. As a positive control, 3.0 µL of Nassau grouper DNA was used. Negative control was PCR-grade water. The PCR products were visualized on agarose gel then the PCR product was sequenced (MCLAB) to confirm the identity of the target species within the eDNA spiked samples.

## Results

### Oceanic physical data

The oceanic conditions at three of the four aggregation sites were monitored during the entire sampling duration (30–40 days). The exception was Bajo de Sico which presented an unfavorable geomorphology (*i.e.,* no flat surfaces around the FSA site core) for securing the AWAC. At the other three locations, the current velocity and direction were charted and available for analysis at the exact time of each sampling event. At Abrir la Sierra, on the peak day of spawning, conditions indicated 0.3–0.4 m/s current velocity in a northerly direction during the time of sampling (Fig. S1). At Mona Island, conditions indicated 0.8–0.9 m/s current velocity in a southeasterly direction during the time of sampling (Fig. S2). At La Parguera, conditions indicated 0.1–0.2 m/s current velocity in a westerly direction during the time of sampling (Fig. S3).

### Primer development

The CO1 primers were only designed for three of the four target species (red hind, Nassau grouper, and mutton snapper) with a size of 285 bp and the 12S primers were designed with a 100 bp size for each of the four species. Primers were tested within and between species. Each set of primers only amplified the target species for that primer, as evidenced through gel electrophoresis. There was no unwanted amplification between primers and thus the primers were successful at only amplifying the target organism.

### Traditional PCR

After primer specificity was confirmed, traditional PCRs were performed with species-specific primers on eDNA samples from each respective aggregation. Total DNA concentrations in the eDNA samples ranged from 0.2ng/µL to 88.2ng/µL (Table S1). Most of the PCR products were deemed immediately unsuccessful (no amplification, smear bands). Changes were made in the protocol’s initial denaturing time, annealing time, extension time and the number of amplification cycles (wherein we either increased or decreased these values, Table S2) and faint bands and smears were eventually observed but ultimately these changes failed to provide positive amplification, as further evidenced through failed sequencing.

### Quantitative PCR

Samples also underwent real-time PCR (qPCR). Series dilution and melt curves illustrated the need for different reaction conditions for each species primer set. Values showed the number of cycles needed to reach fluorescence for each species using 12S species-specific primers (Table 3).

**Table 3:** qPCR optimal temperature and Cq values for each species-specific primer set for the 12S marker.

Species	Optimal temperature	Cq
Red hind	55.8 °C	20.21
Nassau grouper	58.5 °C	16.53
Yellowfin grouper	54.3 °C	20.10
Mutton snapper	51.8 °C	18.16

All eDNA samples had different amplifications that started with the positive control with 16.78 Cq, followed by the NTCs with 26.21 Cq, and lastly eDNA samples derived from sediments for 29.55 Cq. No negative control samples were amplified. There was also a double peak observed during the melt curve. Samples were run on 1.5% agarose gel and positive bands were visualized under UV light; however, bands appeared to be unspecific smears, and some were double bands indicating primer dimers or unspecific amplification. Amplification efficiency was found to be higher than 110% in most cases.

Two mutton snapper eDNA samples showed positive detection of the target species with a Ct of 30, which matched to an NCBI reference (GenBank accession # EF095569.1, # OP591353). For the yellowfin eDNA samples, there were four positive amplifications yet only one sample sequenced successfully with a 100% match to NCBI reference database (GenBank accession # AY949419.1). Thus, although positive results were obtained, they were inconsistent.

To cross-validate these results, these eDNA samples were also tested by a third-party (Laragen Inc, USA) using qPCR. Similar results were obtained, including amplification of NTC. All samples that amplified with a determined Ct were sequenced and tested against NCBI database, but only one Yellowfin water sample resulted in a 100% match (GenBank accession # AY949419.1). Thus, both in-house and third-party validated qPCR processing indicated low efficiency and inability to generate consistent results among sampling methods and within aggregation replicates.

### Spiking the eDNA samples

After unsuccessful PCR and qPCR attempts on the eDNA samples, despite extensive primer development and QAQC, an attempt was then made to determine if the target species’ DNA quantity was simply too low to be detected from the environmental samples. Thus, traditional PCR using Nassau grouper DNA inserted into eDNA Nassau samples provided a positive result with the CO1 marker. Spiked samples amplified successfully while samples without the addition of tissue DNA did not amplify. The eDNA samples which had general DNA concentrations of 5.0 ng/µL (D), 6.1 ng/µL(V) and 3.0 ng/µL(S) were used to perform the spiked PCR.

These samples were spiked with 0.25 µL (19.75 ng/µL), 0.5 (39.5 ng/µL), 0.75 (59.25 ng/µL) and 1.0 µL (79 ng/µL) of Nassau grouper tissue DNA in a reaction volume of 25 µL. Samples spiked with 0.5–1 µL amplified successfully. PCR products were sequenced (MCLAB, California) to confirm identity which successfully matched with the Nassau grouper voucher (GenBank Accession # JQ841568.1).

Thus, the lowest limit of detection was determined to be 39.5 ng/µL of Nassau DNA. The ability to detect the target fish within the spiked samples at least indicates that there were no flaws with the primer design or sampling methodology. At the same time of sampling, the maximum count of Nassau groupers was estimated at 58 individuals.

## Discussion

Working in an open ocean tropical system proved to be a difficult task for single-species detection from eDNA sampling. A recent study determined that the concentration of eDNA and the overall DNA yield was higher in semi-closed environments such as estuaries compared to open environments such as offshore and clear water habitats (Kumar et al., 2022). Furthermore, the persistence of DNA can vary between the sampling methods, where water is found to have higher degradation rates compared to sediment (Sakata et al., 2021). Given these limitations, our study made a significant effort to focus sampling at the exact known times and places of FSA formation and using a variety of sampling methods to obtain the highest DNA yield as possible. In addition, specific primers were designed to amplify our target species, which in a priori testing proved to effectively detect only the target species of interest. Despite these controlled factors, both traditional PCR and qPCR proved to be ineffective at detecting target species DNA in our eDNA samples.

Sensitivity to detect a target individual or group of individuals greatly depends on the dispersion of target DNA in the sampling site (Furlan et al., 2016). In our project, the use of the AWAC provided a retrospective look at what occurred during our exact times of sampling. For example, at the yellowfin aggregation, the current velocity was roughly two knots from the surface to the depth of sampling. Thus, water currents could have quickly dispersed and reduced our target DNA concentrations, despite sampling during high abundances (relative) of target fish and at the known peak spawning times where the fish are found to aggregate close together. Additionally, factors such as UV radiation disturb DNA base pair bonds, influencing and reducing eDNA persistence throughout the water column (Pilliod et al., 2014) and warm temperatures, such as those in the tropics, are known to exacerbate eDNA degradation rates (Dejean et al., 2011). Higher water temperatures promote fish mobility and metabolism (Petty et al., 2012; Xu, Letcher & Nislow, 2010) increasing the rate at which an organism sheds DNA. Higher temperatures then could be associated with higher DNA shedding rates but simultaneously reduce DNA concentrations altogether. This could account for the variation in our eDNA sample concentrations, where general DNA concentration varied widely from 0.2 ng/µL to 88.2 ng/µL between samples and sampling methods.

Lack of amplification and/or negative results could also be false negatives. False negatives are known as undetected taxa that are actually present (Ficetola et al., 2015) and can start to occur within 48 h of sampling since 90% of eDNA concentration degrades in the first two days (Forsström & Vasemägi, 2016; Sassoubre et al., 2016). Most of the PCR products were deemed immediately unsuccessful (no amplification, smear bands) or were found to be erroneous after validation through sequencing indicated non-specific amplification despite valid visual representation on the gel (*e.g.*, the size of the amplified band was of the expected size). False negatives occur when low amounts of target DNA are actually present in the eDNA water sample, but the results fall below needed detection levels (Rees et al., 2014). This phenomenon can occur despite the target organism or group of organisms being present during sampling (Ficetola et al., 2008). However, DNA belonging to non-target species can exacerbate the chance of false negatives (Rees et al., 2014) which is an uncontrollable factor in this study’s open ocean system. In our study, the target species was observed at the time of sampling for three out of our four aggregations, and samples were often collected directly under or next to the target species itself. So, it can be implied that DNA shed from these three targeted species was collected. Even so, if the quantity of DNA collected falls below the detection level, no detection would be possible.

Takahara et al. (2012) found that the abundance of a target fish species could be measured by the quantity of eDNA, and conversely Kelly et al. (2014) found that the eDNA sequences are directly related to the relative abundance of a target population. In our study, low individual count—albeit high compared to non-spawning times—could have resulted in low concentrations of target species DNA and overall scarce eDNA yield. These results were confirmed after performing a traditional PCR with Nassau grouper eDNA samples in which these samples were spiked with DNA from Nassau grouper tissue extraction. Since the species-specific primers were able to successfully amplify the target DNA from the eDNA samples, we can conclude that our primer set was valid, and our sampling methodology was not hindering the ability to detect the target fish.

The maximum fish abundance observed on our peak sampling days was 32 red hind, 58 Nassau grouper, 45 yellowfin and no mutton snapper were observed at their FSA. These numbers are low compared to previously documented counts, such as for red hind which contained 282 individuals (Shapiro, Sadovy & McGehee, 1993; abundance calculated by converting their density to our survey area). Other spawning aggregations are known to range from 100s to 1,000s (Nemeth, 2005; Sadovy de Mitcheson et al., 2008; Sadovy de Mitcheson & Colin, 2011), such as the Cayman Islands for Nassau grouper (Whaylen et al., 2004). In other words, compared with past aggregations or those in other neighboring islands, the number of individuals documented during this study was exponentially lower. Similar studies focused on fish spawning aggregations also use zooplankton nets to collect ichthyoplankton as well as eDNA water samples to compare and correlate detection rates to oocytes and spermatozoa present (Bylemans et al., 2017; Hayer et al., 2020). Spermatozoa are small and abundant in the water column during spawning events which makes them a major source of eDNA during spawning. Gamete release is directly related to peaks in eDNA concentration and overall DNA abundance and detection (Tsuji & Shibata, 2021), and spermatozoa specifically remain in the water column (Bylemans et al., 2017). However, in our case, the exact time of fish spawning is unknown mdash and has never been observed at any of these FSAs—but is estimated to occur at the low light hours of the crepuscular evening, when conditions at sea are unfavorable for diving and navigating these aggregation sites.

## Conclusions

Environmental DNA has the potential to become a promising tool for the study of marine fish spawning aggregations in the Caribbean. However, open oceans present numerous uncontrolled variables that amplify the degradation rates of DNA. For single species detection, the target species DNA must be in detectable concentrations in these open systems which are largely based on the number of target individuals present. For future eDNA studies, it is imperative to take into consideration the importance of species abundance for eDNA yield and species detection.

##  Supplemental Information

10.7717/peerj.14810/supp-1 Supplemental Information 1 Hydrodynamic conditions during sampling at Abrir la Sierra Top panel: Water depth (blue line) and water temperature (orange line) measured by the AWAC; gray rectangles denote times of dives and/or water samples. Middle panel: Direction of depth-averaged current; gray rectangles denote times of dives and/or water samples. Bottom panel: Velocity magnitude as a function of time ( *x*-axis) and distance ( *y*-axis) from the bottom-mounted AWAC sensor; colors represent current speed in meters per second.

10.7717/peerj.14810/supp-2 Supplemental Information 2 Hydrodynamic conditions during sampling at Mona Island Top panel: Water depth (blue line) and water temperature (orange line) measured by the AWAC; gray rectangles denote times of dives and/or water samples. Middle panel: Direction of depth-averaged current; gray rectangles denote times of dives and/or water samples. Bottom panel: Velocity magnitude as a function of time ( *x*-axis) and distance ( *y*-axis) from the bottom-mounted AWAC sensor; colors represent current speed in meters per second.

10.7717/peerj.14810/supp-3 Supplemental Information 3 Hydrodynamic conditions during sampling in La Parguera Top panel: Water depth (blue line) and water temperature (orange line) measured by the AWAC; gray rectangles denote times of dives and/or water samples. Middle panel: Direction of depth-averaged current; gray rectangles denote times of dives and/or water samples. Bottom panel: Velocity magnitude as a function of time ( *x*-axis) and distance ( *y*-axis) from the bottom-mounted AWAC sensor; colors represent current speed in meters per second.

10.7717/peerj.14810/supp-4 Supplemental Information 4 The total DNA concentrations of the samples analyzed in this study D represents Diver collected sample, N is the VanDoorn sample, S is the sediment sample, OS is ocean surface and D is distilled water, CD is control diver, CN is control VanDoorn and CS is control sediment.

10.7717/peerj.14810/supp-5 Supplemental Information 5 A list of PCR trials conducted during this study indicating the various protocol changes utilized to attempt to amplify the target DNA Under Protocol section, Denat. = denaturing, Ann. = annealing, Exten. = extension and Final Exten.= final extension. In Sample ID, the Neg Cont = negative control and Pos Cont= positive control.

## Additional Information and Declarations

### Field Study Permissions

The following information was supplied relating to field study approvals (i.e., approving body and any reference numbers): All sample collection, equipment deployment, and monitoring were conducted under a local permit (DRNA scientific permit #2018-IC-075).

### DNA Deposition

The following information was supplied regarding the deposition of DNA sequences: Sequences are available at GenBank: OP591353.

### Data Availability

The following information was supplied regarding data availability: The DNA concentrations of each eDNA sample is available in the Supplemental Files.
