# Supplementary figures and images for: Using eDNA sampling for species-specific fish detection in tropical oceanic samples: limitations and recommendations for future use
Source: PeerJ. 2023 Feb 2;11:e14810. doi: 10.7717/peerj.14810 (PMC9899429; doi:10.7717/peerj.14810)

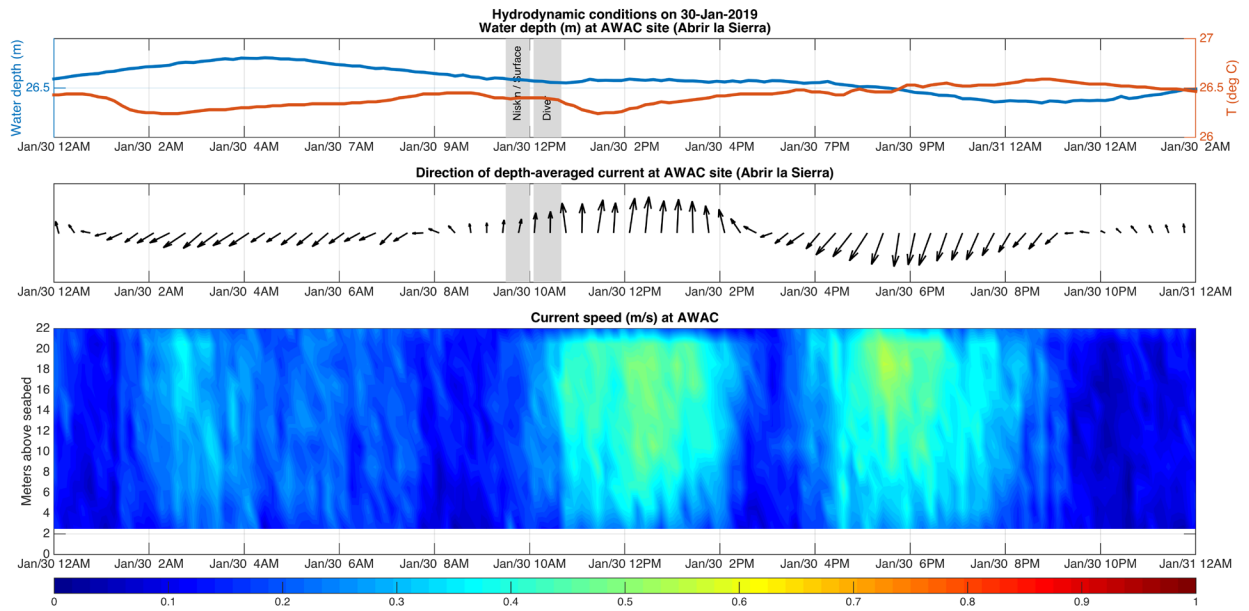

Supplement: Supplemental Information 1 — Top panel: Water depth (blue line) and water temperature (orange line) measured by the AWAC; gray rectangles denote times of dives and/or water samples. Middle panel: Direction of depth-averaged current; gray rectangles denote times of dives and/or water samples. Bottom panel: Velocity magnitude as a function of time ( x-axis) and distance ( y-axis) from the bottom-mounted AWAC sensor; colors represent current speed in meters per second. [file peerj-11-14810-s001.pdf]

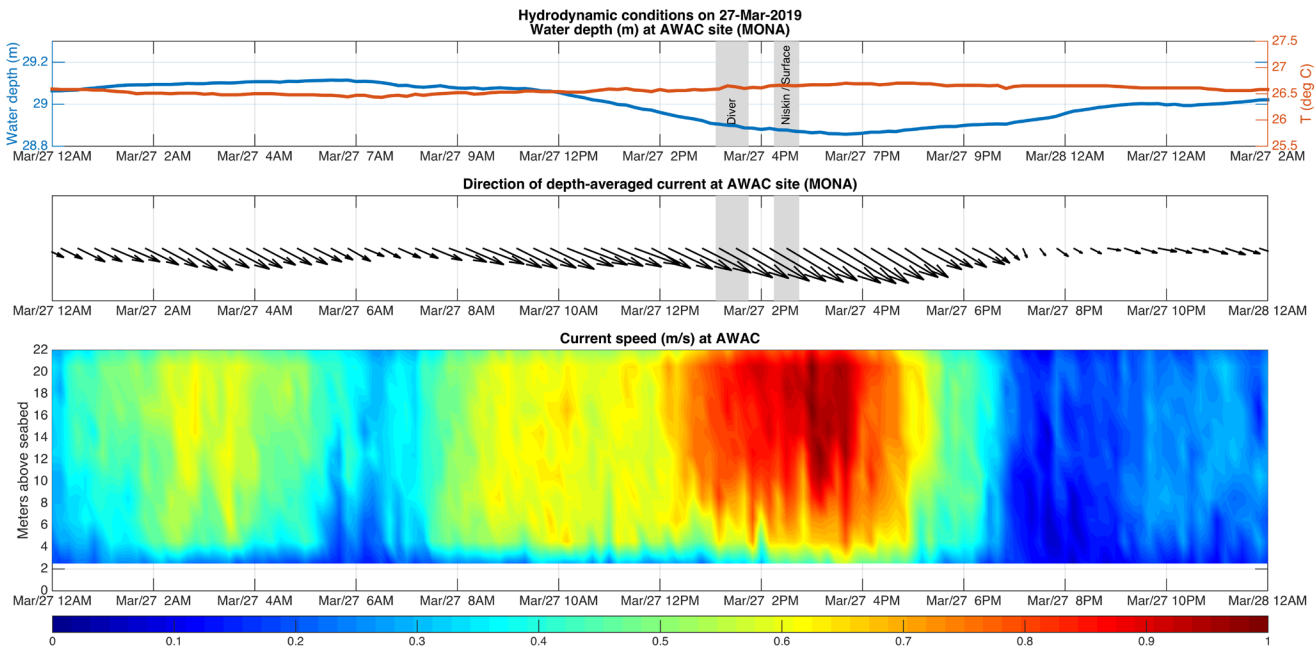

Supplement: Supplemental Information 2 — Top panel: Water depth (blue line) and water temperature (orange line) measured by the AWAC; gray rectangles denote times of dives and/or water samples. Middle panel: Direction of depth-averaged current; gray rectangles denote times of dives and/or water samples. Bottom panel: Velocity magnitude as a function of time ( x-axis) and distance ( y-axis) from the bottom-mounted AWAC sensor; colors represent current speed in meters per second. [file peerj-11-14810-s002.pdf]

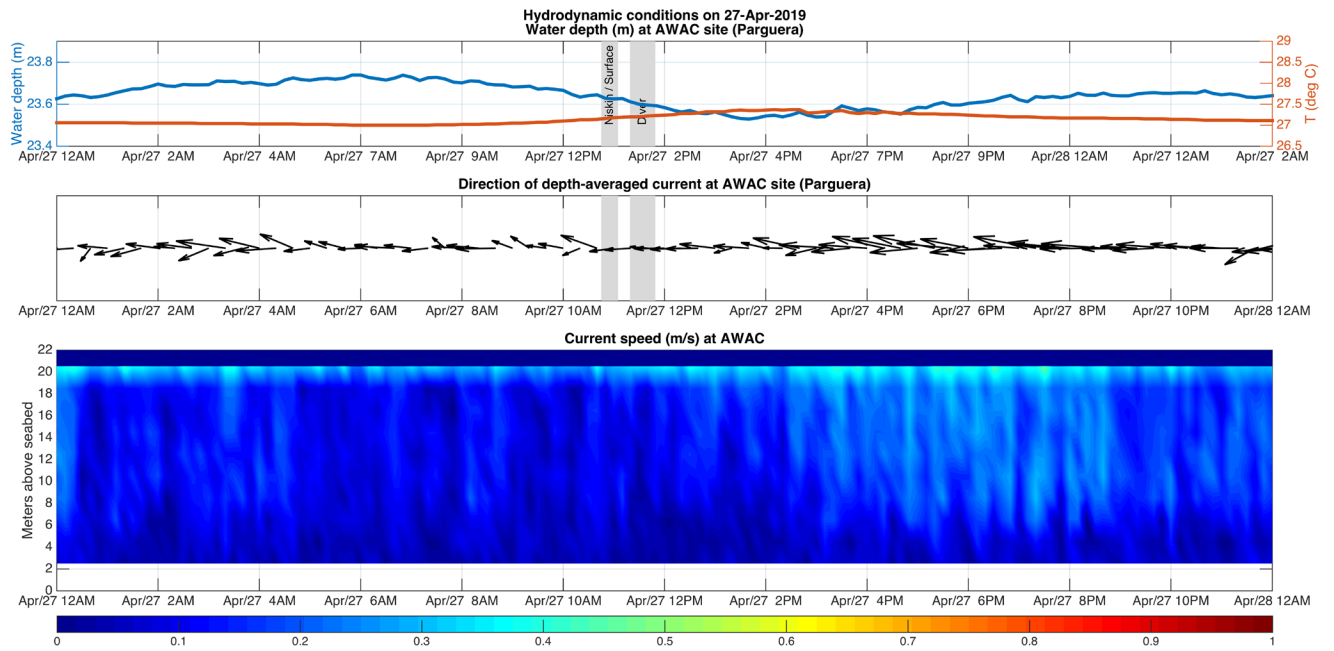

Supplement: Supplemental Information 3 — Top panel: Water depth (blue line) and water temperature (orange line) measured by the AWAC; gray rectangles denote times of dives and/or water samples. Middle panel: Direction of depth-averaged current; gray rectangles denote times of dives and/or water samples. Bottom panel: Velocity magnitude as a function of time ( x-axis) and distance ( y-axis) from the bottom-mounted AWAC sensor; colors represent current speed in meters per second. [file peerj-11-14810-s003.pdf]
