# Supplementary material for: Using eDNA sampling for species-specific fish detection in tropical oceanic samples: limitations and recommendations for future use
Source: PeerJ. 2023 Feb 2;11:e14810. doi: 10.7717/peerj.14810 (PMC9899429; doi:10.7717/peerj.14810)
Supplement: Supplemental Information 4 — D represents Diver collected sample, N is the VanDoorn sample, S is the sediment sample, OS is ocean surface and D is distilled water, CD is control diver, CN is control VanDoorn and CS is control sediment. [file peerj-11-14810-s004.docx]

| Red hind | | Nassau grouper | | Yellowfin grouper | | | Mutton snapper | | |
| --- | --- | --- | --- | --- | --- | --- | --- | --- | --- |
| Sample | **(ng/µL)** | **Sample** | **(ng/µL)** | **Sample** | **(ng/µL)** | | **Sample** | **(ng/µL)** | |
| D1 | 54.5 | **D1** | 10 | **D1** | | 60 | **D1** | | 15.7 |
| D2 | 88.2 | **D2** | 7.4 | **D2** | | 2.8 | **D2** | | 19.0 |
| D3 | 25.1 | **D3** | 7.0 | **D3** | | 4.9 | **D3** | | 7.8 |
| N1 | 37.8 | **N1** | 6.1 | **N1** | | 6.3 | **N1** | | 14.7 |
| N2 | 19.6 | **N2** | 6.9 | **N2** | | 4.7 | **N2** | | 13.6 |
| N3 | 16.5 | **N3** | 5.4 | **N3** | | 4.6 | **N3** | | 9.2 |
| S1 | 3.8 | **S1** | 6.1 | **S1** | | 8.9 | **S1** | | 12.7 |
| S2 | 7.8 | **S2** | 6.1 | **S2** | | 3.6 | **S2** | | 12.1 |
| S3 | 2.3 | **S3** | 5.9 | **S3** | | 2.0 | **S3** | | 1.7 |
| OS1 | 15.1 | **OS1** | 7.8 | **OS1** | | 11.2 | **OS1** | | 28.6 |
| OS2 | 11.0 | **OS2** | 4.0 | **OS2** | | 8.2 | **OS2** | | 13.1 |
| OS3 | 16.4 | **OS3** | 3.6 | **OS3** | | 12.3 | **OS3** | | 8.0 |
| D1 | 13.7 | **D1** | N/A | **D1** | | 7.1 | **D1** | | 10.4 |
| D2 | 10.1 | **D2** | N/A | **D2** | | 4.9 | **D2** | | 49.0 |
| D3 | 16.9 | **D3** | 5.0 | **D3** | | 5.5 | **D3** | | 19.3 |
| N1 | 16.2 | **N1** | N/A | **N1** | | 8.1 | **N1** | | 21.6 |
| N2 | 15.2 | **N2** | N/A | **N2** | | 8.0 | **N2** | | 10.0 |
| N3 | 24.8 | **N3** | N/A | **N3** | | 8.2 | **N3** | | 12.1 |
| S1 | 2.0 | **S1** | 8.0 | **S1** | | 4.9 | **S1** | | 0.5 |
| S2 | 3.4 | **S2** | N/A | **S2** | | 7.3 | **S2** | | 6.2 |
| S3 | 1.8 | **S3** | N/A | **S3** | | 2.2 | **S3** | | 8.8 |
| OS1 | 15.2 | **OS1** | N/A | **OS1** | | 5.8 | **OS1** | | 26.1 |
| OS2 | 4.2 | **OS2** | 0.8 | **OS2** | | 10.1 | **OS2** | | 30.6 |
| OS3 | 21.1 | **OS3** | 2.5 | **OS3** | | 4.2 | **OS3** | | 14.1 |
| CD1 | 11.5 | **CD1** | 1.3 | **CD1** | | 9.4 | **CD1** | | 35.4 |
| CD2 | 12.7 | **CD2** | 0.6 | **CD2** | | 5.6 | **CD2** | | 29.1 |
| CD3 | 7.4 | **CD3** | 2.7 | **CD3** | | 8.8 | **CD3** | | 16.8 |
| CN1 | 20.9 | **CN1** | 0.5 | **CN1** | | 46.2 | **CN1** | | 14.1 |
| CN2 | 6.0 | **CN2** | 0.1 | **CN2** | | 9.4 | **CN2** | | 25.1 |
| CN3 | 12.3 | **CN3** | 0.9 | **CN3** | | 8.0 | **CN3** | | 43.1 |
| CS1 | 2.3 | **CS1** | 4.7 | **CS1** | | 7.1 | **CS1** | | 1.3 |
| CS2 | 1.7 | **CS2** | 0.8 | **CS2** | | 4.1 | **CS2** | | 15.4 |
| CS3 | 0.5 | **CS3** | 6.2 | **CS3** | | 3.1 | **CS3** | | 2.4 |
| D1 | 8.3 | **D1** | 19.9 | **D1** | | 9.4 | **D1** | | 35.4 |
| D2 | 34.7 | **D2** | 11.1 | **D2** | | 5.6 | **D2** | | 29.1 |
| D3 | 12.7 | **D3** | 13.9 | **D3** | | 8.8 | **D3** | | 16.8 |
| N1 | 3.0 | **N1** | 13.7 | **N1** | | 46.2 | **N1** | | 14.1 |
| N2 | 4.2 | **N2** | 9.0 | **N2** | | 9.4 | **N2** | | 25.1 |
| N3 | 15.4 | **N3** | 24.1 | **N3** | | 8.0 | **N3** | | 43.1 |
| OS1 | 9.7 | **OS1** | 12.6 | **OS1** | | 2.1 | **OS1** | | 0.3 |
| OS2 | 8.6 | **OS2** | 15.1 | **OS2** | | 2.6 | **OS2** | | 1.2 |
| OS3 | 16.4 | **OS3** | 12.3 | **OS3** | | 0.8 | **OS3** | | 1.9 |
| S1 | 4.9 | **S1** | 3.9 | **S1** | | 7.1 | **S1** | | 1.3 |
| S2 | 8.2 | **S2** | 9.2 | **S2** | | 4.1 | **S2** | | 15.4 |
| S3 | 3.6 | **S3** | 2.0 | **S3** | | 3.1 | **S3** | | 2.4 |
| D1 | 4.1 | **D1** | 1.1 | **D1** | | 2.8 | **D1** | | N/A |
| D2 | 6.1 | **D2** | 5.3 | **D2** | | 4.0 | **D2** | | N/A |
| D3 | 18.2 | **D3** | 4.1 | **D3** | | 1.3 | **D3** | | N/A |
| N1 | 10.0 | **N1** | 1.6 | **N1** | | -1.2 | **N1** | | N/A |
| N2 | 24.1 | **N2** | 7.4 | **N2** | | 0.8 | **N2** | | N/A |
| N3 | 4.3 | **N3** | 6.0 | **N3** | | -1.8 | **N3** | | N/A |
| OS1 | 1.2 | **OS1** | 2.1 | **OS1** | | 0.9 | **OS1** | | N/A |
| OS2 | 6.2 | **OS2** | 2.8 | **OS2** | | 2.1 | **OS2** | | N/A |
| OS3 | 4.7 | **OS3** | 2.2 | **OS3** | | 1.9 | **OS3** | | N/A |
| S1 | 10.6 | **S1** | 5.8 | **S1** | | 5.6 | **S1** | | N/A |
| S2 | 20.2 | **S2** | 7.4 | **S2** | | 9.4 | **S2** | | N/A |
| S3 | 9.7 | **S3** | 6.4 | **S3** | | 4.1 | **S3** | | N/A |
