# Supplementary material for: Using eDNA sampling for species-specific fish detection in tropical oceanic samples: limitations and recommendations for future use
Source: PeerJ. 2023 Feb 2;11:e14810. doi: 10.7717/peerj.14810 (PMC9899429; doi:10.7717/peerj.14810)
Supplement: Supplemental Information 5 — Under Protocol section, Denat. = denaturing, Ann. = annealing, Exten. = extension and Final Exten.= final extension. In Sample ID, the Neg Cont = negative control and Pos Cont= positive control. [file peerj-11-14810-s005.docx]

| **PCR #** | **Sample ID** | **Reaction** | | | | | **Protocol** | | | | | | **Gene** | **Result** |
| --- | --- | --- | --- | --- | --- | --- | --- | --- | --- | --- | --- | --- | --- | --- |
|  |  | **DNA (µL)** | **H2O (µL)** | **Primer F (µL)** | **Primer R (µL)** | **Master**  **Mix (µL)** | **Initial Denat.** | **Denat.** | **Ann.** | **Exten.** | **Final Exten.** | **Cycles** |  |  |
| **1** | D1 - RH | 1 | 11 | 0.2 | 0.2 | 12.5 | 95°C x 3 min | 95°C x 20s | 48.5°C x 30s | 72°C x 1 min | 72°C x 5 min | 25 | CO1 | NEGATIVE |
|  | D2 - RH | 1 | 11 | 0.2 | 0.2 | 12.5 |  |  |  |  |  |  |  | NEGATIVE |
|  | D3 - RH | 1 | 11 | 0.2 | 0.2 | 12.5 |  |  |  |  |  |  |  | NEGATIVE |
|  | N1 -RH | 1 | 11 | 0.2 | 0.2 | 12.5 |  |  |  |  |  |  |  | NEGATIVE |
|  | N2 -RH | 1 | 11 | 0.2 | 0.2 | 12.5 |  |  |  |  |  |  |  | NEGATIVE |
|  | N3 - RH | 1 | 11 | 0.2 | 0.2 | 12.5 |  |  |  |  |  |  |  | NEGATIVE |
|  | OC1 - RH | 1 | 11 | 0.2 | 0.2 | 12.5 |  |  |  |  |  |  |  | NEGATIVE |
|  | Neg Cont | 1 | 11 | 0.2 | 0.2 | 12.5 |  |  |  |  |  |  |  | N/A |
| **2** | S1 - RH | 1 | 11 | 0.2 | 0.2 | 12.5 | 95°C x 3 min | 95°C x 10s | 49.9°C x 30s | 72°C x 1 min | 72°C x 5 min | 25 | CO1 | NEGATIVE |
|  | D1 - RH | 1 | 11 | 0.2 | 0.2 | 12.5 |  |  |  |  |  |  |  | NEGATIVE |
|  | D2 - RH | 1 | 11 | 0.2 | 0.2 | 12.5 |  |  |  |  |  |  |  | NEGATIVE |
|  | D3 - RH | 1 | 11 | 0.2 | 0.2 | 12.5 |  |  |  |  |  |  |  | NEGATIVE |
|  | N1 -RH | 1 | 11 | 0.2 | 0.2 | 12.5 |  |  |  |  |  |  |  | NEGATIVE |
|  | N2 -RH | 1 | 11 | 0.2 | 0.2 | 12.5 |  |  |  |  |  |  |  | NEGATIVE |
|  | N3 - RH | 1 | 11 | 0.2 | 0.2 | 12.5 |  |  |  |  |  |  |  | NEGATIVE |
|  | Neg Cont | 1 | 11 | 0.2 | 0.2 | 12.5 |  |  |  |  |  |  |  | N/A |
| **3** | S1-2-RH | 3 | 9.1 | 0.2 | 0.2 | 12.5 | 95°C x 3 min | 95°C x 10s | 51°C x 30s | 72°C x 1 min | 72°C x 5 min | 25 | CO1 | NEGATIVE |
|  | S2-2-RH | 3 | 9.1 | 0.2 | 0.2 | 12.5 |  |  |  |  |  |  |  | NEGATIVE |
|  | D2-RH | 3 | 9.1 | 0.2 | 0.2 | 12.5 |  |  |  |  |  |  |  | NEGATIVE |
|  | D3-RH | 3 | 9.1 | 0.2 | 0.2 | 12.5 |  |  |  |  |  |  |  | NEGATIVE |
|  | N2-RH | 3 | 9.1 | 0.2 | 0.2 | 12.5 |  |  |  |  |  |  |  | NEGATIVE |
|  | N3-2-RH | 3 | 9.1 | 0.2 | 0.2 | 12.5 |  |  |  |  |  |  |  | NEGATIVE |
|  | OS1-RH | 3 | 9.1 | 0.2 | 0.2 | 12.5 |  |  |  |  |  |  |  | NEGATIVE |
|  | Neg Cont | 3 | 9.1 | 0.2 | 0.2 | 12.5 |  |  |  |  |  |  |  | N/A |
| **4** | S1-RH | 1 | 11 | 0.2 | 0.2 | 12.5 | 95°C x 3 min | 95°C x 10s | 51°C x 30s | 72°C x 1 min | 72°C x 1 min | 30 | CO1 | NEGATIVE |
|  | S2-RH | 1 | 11 | 0.2 | 0.2 | 12.5 |  |  |  |  |  |  |  | NEGATIVE |
|  | S3-RH | 1 | 11 | 0.2 | 0.2 | 12.5 |  |  |  |  |  |  |  | NEGATIVE |
|  | D1-RH | 1 | 11 | 0.2 | 0.2 | 12.5 |  |  |  |  |  |  |  | NEGATIVE |
|  | D2-RH | 1 | 11 | 0.2 | 0.2 | 12.5 |  |  |  |  |  |  |  | NEGATIVE |
|  | D3-RH | 1 | 11 | 0.2 | 0.2 | 12.5 |  |  |  |  |  |  |  | NEGATIVE |
|  | N1-RH | 1 | 11 | 0.2 | 0.2 | 12.5 |  |  |  |  |  |  |  | NEGATIVE |
|  | N2-RH | 1 | 11 | 0.2 | 0.2 | 12.5 |  |  |  |  |  |  |  | NEGATIVE |
|  | N3-RH | 1 | 11 | 0.2 | 0.2 | 12.5 |  |  |  |  |  |  |  | NEGATIVE |
|  | OC1-RH | 1 | 11 | 0.2 | 0.2 | 12.5 |  |  |  |  |  |  |  | NEGATIVE |
|  | OC2-RH | 1 | 11 | 0.2 | 0.2 | 12.5 |  |  |  |  |  |  |  | NEGATIVE |
|  | OC3-RH | 1 | 11 | 0.2 | 0.2 | 12.5 |  |  |  |  |  |  |  | NEGATIVE |
|  | DC1-RH | 1 | 11 | 0.2 | 0.2 | 12.5 |  |  |  |  |  |  |  | NEGATIVE |
|  | DC2-RH | 1 | 11 | 0.2 | 0.2 | 12.5 |  |  |  |  |  |  |  | NEGATIVE |
|  | DC3-RH | 1 | 11 | 0.2 | 0.2 | 12.5 |  |  |  |  |  |  |  | NEGATIVE |
|  | Neg Cont | 1 | 11 | 0.2 | 0.2 | 12.5 |  |  |  |  |  |  |  | N/A |
| **5** | S1-RH | 2 | 10 | 0.2 | 0.2 | 12.5 | 95°C x 3 min | 95°C x 10s | 51°C x 30s | 72°C x 1 min | 72°C x 2 min | 35 | CO1 | NEGATIVE |
|  | S2-RH | 2 | 10 | 0.2 | 0.2 | 12.5 |  |  |  |  |  |  |  | NEGATIVE |
|  | S3-RH | 2 | 10 | 0.2 | 0.2 | 12.5 |  |  |  |  |  |  |  | NEGATIVE |
|  | D1-RH | 2 | 10 | 0.2 | 0.2 | 12.5 |  |  |  |  |  |  |  | NEGATIVE |
|  | D2-RH | 2 | 10 | 0.2 | 0.2 | 12.5 |  |  |  |  |  |  |  | NEGATIVE |
|  | D3-RH | 2 | 10 | 0.2 | 0.2 | 12.5 |  |  |  |  |  |  |  | NEGATIVE |
|  | N1-RH | 2 | 10 | 0.2 | 0.2 | 12.5 |  |  |  |  |  |  |  | NEGATIVE |
|  | N2-RH | 2 | 10 | 0.2 | 0.2 | 12.5 |  |  |  |  |  |  |  | NEGATIVE |
|  | N3-RH | 2 | 10 | 0.2 | 0.2 | 12.5 |  |  |  |  |  |  |  | NEGATIVE |
|  | OC1-RH | 2 | 10 | 0.2 | 0.2 | 12.5 |  |  |  |  |  |  |  | NEGATIVE |
|  | OC2-RH | 2 | 10 | 0.2 | 0.2 | 12.5 |  |  |  |  |  |  |  | NEGATIVE |
|  | OC3-RH | 2 | 10 | 0.2 | 0.2 | 12.5 |  |  |  |  |  |  |  | NEGATIVE |
|  | DC1-RH | 2 | 10 | 0.2 | 0.2 | 12.5 |  |  |  |  |  |  |  | NEGATIVE |
|  | DC2-RH | 2 | 10 | 0.2 | 0.2 | 12.5 |  |  |  |  |  |  |  | NEGATIVE |
|  | DC3-RH | 2 | 10 | 0.2 | 0.2 | 12.5 |  |  |  |  |  |  |  | NEGATIVE |
|  | Neg Cont | 2 | 10 | 0.2 | 0.2 | 12.5 |  |  |  |  |  |  |  | N/A |
| **6** | S1-RH | 3 | 9 | 0.2 | 0.2 | 12.5 | 95°C x 3 min | 95°C x 10s | 51°C x 30s | 72°C x 1 min | 72°C x 2 min | 35 | CO1 | NEGATIVE |
|  | S2-RH | 3 | 9 | 0.2 | 0.2 | 12.5 |  |  |  |  |  |  |  | NEGATIVE |
|  | S3-RH | 3 | 9 | 0.2 | 0.2 | 12.5 |  |  |  |  |  |  |  | NEGATIVE |
|  | D1-RH | 3 | 9 | 0.2 | 0.2 | 12.5 |  |  |  |  |  |  |  | NEGATIVE |
|  | D2-RH | 3 | 9 | 0.2 | 0.2 | 12.5 |  |  |  |  |  |  |  | NEGATIVE |
|  | D3-RH | 3 | 9 | 0.2 | 0.2 | 12.5 |  |  |  |  |  |  |  | NEGATIVE |
|  | N1-RH | 3 | 9 | 0.2 | 0.2 | 12.5 |  |  |  |  |  |  |  | NEGATIVE |
|  | N2-RH | 3 | 9 | 0.2 | 0.2 | 12.5 |  |  |  |  |  |  |  | NEGATIVE |
|  | N3-RH | 3 | 9 | 0.2 | 0.2 | 12.5 |  |  |  |  |  |  |  | NEGATIVE |
|  | OC1-RH | 3 | 9 | 0.2 | 0.2 | 12.5 |  |  |  |  |  |  |  | NEGATIVE |
|  | OC2-RH | 3 | 9 | 0.2 | 0.2 | 12.5 |  |  |  |  |  |  |  | NEGATIVE |
|  | OC3-RH | 3 | 9 | 0.2 | 0.2 | 12.5 |  |  |  |  |  |  |  | NEGATIVE |
|  | DC1-RH | 3 | 9 | 0.2 | 0.2 | 12.5 |  |  |  |  |  |  |  | NEGATIVE |
|  | DC2-RH | 3 | 9 | 0.2 | 0.2 | 12.5 |  |  |  |  |  |  |  | NEGATIVE |
|  | DC3-RH | 3 | 9 | 0.2 | 0.2 | 12.5 |  |  |  |  |  |  |  | NEGATIVE |
|  | Neg Cont | 3 | 9 | 0.2 | 0.2 | 12.5 |  |  |  |  |  |  |  | N/A |
| **7** | S1-3-RH | 3 | 9 | 0.2 | 0.2 | 12.5 | 95°C x 3 min | 95°C x 10s | 52°C x 30s | 72°C x 1 min | 72°C x 3 min | 35 | CO1 | NEGATIVE |
|  | S2-3-RH | 3 | 9 | 0.2 | 0.2 | 12.5 |  |  |  |  |  |  |  | NEGATIVE |
|  | S3-3-RH | 3 | 9 | 0.2 | 0.2 | 12.5 |  |  |  |  |  |  |  | NEGATIVE |
|  | D1-3-RH | 3 | 9 | 0.2 | 0.2 | 12.5 |  |  |  |  |  |  |  | NEGATIVE |
|  | D2-3-RH | 3 | 9 | 0.2 | 0.2 | 12.5 |  |  |  |  |  |  |  | NEGATIVE |
|  | D3-3-RH | 3 | 9 | 0.2 | 0.2 | 12.5 |  |  |  |  |  |  |  | NEGATIVE |
|  | N1-3-RH | 3 | 9 | 0.2 | 0.2 | 12.5 |  |  |  |  |  |  |  | NEGATIVE |
|  | N2-3-RH | 3 | 9 | 0.2 | 0.2 | 12.5 |  |  |  |  |  |  |  | NEGATIVE |
|  | N3-3-RH | 3 | 9 | 0.2 | 0.2 | 12.5 |  |  |  |  |  |  |  | NEGATIVE |
|  | OC1-3-RH | 3 | 9 | 0.2 | 0.2 | 12.5 |  |  |  |  |  |  |  | NEGATIVE |
|  | OC2-3-RH | 3 | 9 | 0.2 | 0.2 | 12.5 |  |  |  |  |  |  |  | NEGATIVE |
|  | OC3-3-RH | 3 | 9 | 0.2 | 0.2 | 12.5 |  |  |  |  |  |  |  | NEGATIVE |
|  | DC1-3-RH | 3 | 9 | 0.2 | 0.2 | 12.5 |  |  |  |  |  |  |  | NEGATIVE |
|  | DC2-3-RH | 3 | 9 | 0.2 | 0.2 | 12.5 |  |  |  |  |  |  |  | NEGATIVE |
|  | DC3-3-RH | 3 | 9 | 0.2 | 0.2 | 12.5 |  |  |  |  |  |  |  | NEGATIVE |
|  | Neg Cont | 3 | 9 | 0.2 | 0.2 | 12.5 |  |  |  |  |  |  |  | N/A |
| **8** | S1-3-RH | 5 | 9 | 0.2 | 0.2 | 12.5 | 95°C x 3 min | 95°C x 10s | 52°C x 30s | 72°C x 1 min | 72°C x 3 min | 35 | CO1 | NEGATIVE |
|  | S2-3-RH | 5 | 9 | 0.2 | 0.2 | 12.5 |  |  |  |  |  |  |  | NEGATIVE |
|  | S3-3-RH | 5 | 9 | 0.2 | 0.2 | 12.5 |  |  |  |  |  |  |  | NEGATIVE |
|  | D1-3-RH | 5 | 9 | 0.2 | 0.2 | 12.5 |  |  |  |  |  |  |  | NEGATIVE |
|  | D2-3-RH | 5 | 9 | 0.2 | 0.2 | 12.5 |  |  |  |  |  |  |  | NEGATIVE |
|  | D3-3-RH | 5 | 9 | 0.2 | 0.2 | 12.5 |  |  |  |  |  |  |  | NEGATIVE |
|  | N1-3-RH | 5 | 9 | 0.2 | 0.2 | 12.5 |  |  |  |  |  |  |  | NEGATIVE |
|  | N2-3-RH | 5 | 9 | 0.2 | 0.2 | 12.5 |  |  |  |  |  |  |  | NEGATIVE |
|  | N3-3-RH | 5 | 9 | 0.2 | 0.2 | 12.5 |  |  |  |  |  |  |  | NEGATIVE |
|  | OC1-3-RH | 5 | 9 | 0.2 | 0.2 | 12.5 |  |  |  |  |  |  |  | NEGATIVE |
|  | OC2-3-RH | 5 | 9 | 0.2 | 0.2 | 12.5 |  |  |  |  |  |  |  | NEGATIVE |
|  | OC3-3-RH | 5 | 9 | 0.2 | 0.2 | 12.5 |  |  |  |  |  |  |  | NEGATIVE |
|  | DC1-3-RH | 5 | 9 | 0.2 | 0.2 | 12.5 |  |  |  |  |  |  |  | NEGATIVE |
|  | DC2-3-RH | 5 | 9 | 0.2 | 0.2 | 12.5 |  |  |  |  |  |  |  | NEGATIVE |
|  | DC3-3-RH | 5 | 9 | 0.2 | 0.2 | 12.5 |  |  |  |  |  |  |  | NEGATIVE |
|  | Neg Cont | 5 | 9 | 0.2 | 0.2 | 12.5 |  |  |  |  |  |  |  | N/A |
| **9** | S1-2-NS | 1 | 11 | 0.2 | 0.2 | 12.5 | 95°C x 3 min | 95°C x 10s | 52.6°C x 30s | 72°C x 1 min | 72°C x 3 min | 35 | CO1 | NEGATIVE |
|  | S2-2-NS | 1 | 11 | 0.2 | 0.2 | 12.5 |  |  |  |  |  |  |  | NEGATIVE |
|  | S3-2-NS | 1 | 11 | 0.2 | 0.2 | 12.5 |  |  |  |  |  |  |  | NEGATIVE |
|  | D1-2-NS | 1 | 11 | 0.2 | 0.2 | 12.5 |  |  |  |  |  |  |  | NEGATIVE |
|  | D2-2-NS | 1 | 11 | 0.2 | 0.2 | 12.5 |  |  |  |  |  |  |  | NEGATIVE |
|  | D3-2-NS | 1 | 11 | 0.2 | 0.2 | 12.5 |  |  |  |  |  |  |  | NEGATIVE |
|  | N1-2-NS | 1 | 11 | 0.2 | 0.2 | 12.5 |  |  |  |  |  |  |  | NEGATIVE |
|  | N2-2-NS | 1 | 11 | 0.2 | 0.2 | 12.5 |  |  |  |  |  |  |  | NEGATIVE |
|  | N3-2-NS | 1 | 11 | 0.2 | 0.2 | 12.5 |  |  |  |  |  |  |  | NEGATIVE |
|  | OC1-2-NS | 1 | 11 | 0.2 | 0.2 | 12.5 |  |  |  |  |  |  |  | NEGATIVE |
|  | OC2-2-NS | 1 | 11 | 0.2 | 0.2 | 12.5 |  |  |  |  |  |  |  | NEGATIVE |
|  | OC3-2-NS | 1 | 11 | 0.2 | 0.2 | 12.5 |  |  |  |  |  |  |  | NEGATIVE |
|  | DC1-2-NS | 1 | 11 | 0.2 | 0.2 | 12.5 |  |  |  |  |  |  |  | NEGATIVE |
|  | DC2-2-NS | 1 | 11 | 0.2 | 0.2 | 12.5 |  |  |  |  |  |  |  | NEGATIVE |
|  | DC3-2-NS | 1 | 11 | 0.2 | 0.2 | 12.5 |  |  |  |  |  |  |  | NEGATIVE |
|  | Neg Cont | 1 | 11 | 0.2 | 0.2 | 12.5 |  |  |  |  |  |  |  | N/A |
| **10** | S1-2-NS | 2 | 11 | 1 | 1 | 10 | 95°C x 3 min | 95°C x 10s | 52.6°C x 30s | 72°C x 1 min | 72°C x 3 min | 35 | CO1 | NEGATIVE |
|  | S2-2-NS | 2 | 11 | 1 | 1 | 10 |  |  |  |  |  |  |  | NEGATIVE |
|  | S3-2-NS | 2 | 11 | 1 | 1 | 10 |  |  |  |  |  |  |  | NEGATIVE |
|  | D1-2-NS | 2 | 11 | 1 | 1 | 10 |  |  |  |  |  |  |  | NEGATIVE |
|  | D2-2-NS | 2 | 11 | 1 | 1 | 10 |  |  |  |  |  |  |  | NEGATIVE |
|  | D3-2-NS | 2 | 11 | 1 | 1 | 10 |  |  |  |  |  |  |  | NEGATIVE |
|  | N1-2-NS | 2 | 11 | 1 | 1 | 10 |  |  |  |  |  |  |  | NEGATIVE |
|  | N2-2-NS | 2 | 11 | 1 | 1 | 10 |  |  |  |  |  |  |  | NEGATIVE |
|  | N3-2-NS | 2 | 11 | 1 | 1 | 10 |  |  |  |  |  |  |  | NEGATIVE |
|  | OC1-2-NS | 2 | 11 | 1 | 1 | 10 |  |  |  |  |  |  |  | NEGATIVE |
|  | OC2-2-NS | 2 | 11 | 1 | 1 | 10 |  |  |  |  |  |  |  | NEGATIVE |
|  | OC3-2-NS | 2 | 11 | 1 | 1 | 10 |  |  |  |  |  |  |  | NEGATIVE |
|  | DC1-2-NS | 2 | 11 | 1 | 1 | 10 |  |  |  |  |  |  |  | NEGATIVE |
|  | DC2-2-NS | 2 | 11 | 1 | 1 | 10 |  |  |  |  |  |  |  | NEGATIVE |
|  | DC3-2-NS | 2 | 11 | 1 | 1 | 10 |  |  |  |  |  |  |  | NEGATIVE |
|  | Neg Cont | 2 | 11 | 1 | 1 | 10 |  |  |  |  |  |  |  | N/A |
| **11** | S1-RH | 2 | 11 | 1 | 1 | 10 | 95°C x 3 min | 95°C x 10s | 51°C x 30s | 72°C x 1 min | 72°C x 3 min | 40 | CO1 | NEGATIVE |
|  | S2-RH | 2 | 11 | 1 | 1 | 10 |  |  |  |  |  |  |  | NEGATIVE |
|  | S3-RH | 2 | 11 | 1 | 1 | 10 |  |  |  |  |  |  |  | NEGATIVE |
|  | D1-RH | 2 | 11 | 1 | 1 | 10 |  |  |  |  |  |  |  | NEGATIVE |
|  | D2-RH | 2 | 11 | 1 | 1 | 10 |  |  |  |  |  |  |  | NEGATIVE |
|  | D3-RH | 2 | 11 | 1 | 1 | 10 |  |  |  |  |  |  |  | NEGATIVE |
|  | N1-RH | 2 | 11 | 1 | 1 | 10 |  |  |  |  |  |  |  | NEGATIVE |
|  | N2-RH | 2 | 11 | 1 | 1 | 10 |  |  |  |  |  |  |  | NEGATIVE |
|  | N3-RH | 2 | 11 | 1 | 1 | 10 |  |  |  |  |  |  |  | NEGATIVE |
|  | OC1-RH | 2 | 11 | 1 | 1 | 10 |  |  |  |  |  |  |  | NEGATIVE |
|  | OC2-RH | 2 | 11 | 1 | 1 | 10 |  |  |  |  |  |  |  | NEGATIVE |
|  | OC3-RH | 2 | 11 | 1 | 1 | 10 |  |  |  |  |  |  |  | NEGATIVE |
|  | DC1-RH | 2 | 11 | 1 | 1 | 10 |  |  |  |  |  |  |  | NEGATIVE |
|  | DC2-RH | 2 | 11 | 1 | 1 | 10 |  |  |  |  |  |  |  | NEGATIVE |
|  | DC3-RH | 2 | 11 | 1 | 1 | 10 |  |  |  |  |  |  |  | NEGATIVE |
|  | Neg Cont | 2 | 11 | 1 | 1 | 10 |  |  |  |  |  |  |  | N/A |
| **12** | S2-RH | 4 | 1.5 | 1 | 1 | 7.5 | 95°C x 3 min | 95°C x 10s | 51°C x 30s | 72°C x 45s | 72°C x 5 min | 35 | CO1 | NEGATIVE |
|  | D2-RH | 4 | 1.5 | 1 | 1 | 7.5 |  |  |  |  |  |  |  | NEGATIVE |
|  | N2-RH | 4 | 1.5 | 1 | 1 | 7.5 |  |  |  |  |  |  |  | NEGATIVE |
|  | OC2-RH | 4 | 1.5 | 1 | 1 | 7.5 |  |  |  |  |  |  |  | NEGATIVE |
|  | DC2-RH | 4 | 1.5 | 1 | 1 | 7.5 |  |  |  |  |  |  |  | NEGATIVE |
|  | Neg Cont | 4 | 1.5 | 1 | 1 | 7.5 |  |  |  |  |  |  |  | N/A |
| **13** | S3-2-RH | 4 | 1.5 | 1 | 1 | 7.5 | 95°C x 3 min | 95°C x 10s | 52.6°C x 30s | 72°C x 45s | 72°C x 5 min | 35 | CO1 | NEGATIVE |
|  | D3-2-RH | 4 | 1.5 | 1 | 1 | 7.5 |  |  |  |  |  |  |  | NEGATIVE |
|  | N3-2-RH | 4 | 1.5 | 1 | 1 | 7.5 |  |  |  |  |  |  |  | FAINT BAND |
|  | OC3-2-RH | 4 | 1.5 | 1 | 1 | 7.5 |  |  |  |  |  |  |  | NEGATIVE |
|  | DC3-2-RH | 4 | 1.5 | 1 | 1 | 7.5 |  |  |  |  |  |  |  | NEGATIVE |
|  | Neg Cont | 4 | 1.5 | 1 | 1 | 7.5 |  |  |  |  |  |  |  | N/A |
| **14** | S3-2-RH | 4 | 1.5 | 1 | 1 | 7.5 | 95°C x 3 min | 95°C x 10s | 52.6°C x 30s | 72°C x 45s | 72°C x 5 min | 35 | 12S | NEGATIVE |
|  | D3-2-RH | 4 | 1.5 | 1 | 1 | 7.5 |  |  |  |  |  |  |  | NEGATIVE |
|  | N3-2-RH | 4 | 1.5 | 1 | 1 | 7.5 |  |  |  |  |  |  |  | FAINT BAND |
|  | OC3-2-RH | 4 | 1.5 | 1 | 1 | 7.5 |  |  |  |  |  |  |  | NEGATIVE |
|  | DC3-2-RH | 4 | 1.5 | 1 | 1 | 7.5 |  |  |  |  |  |  |  | NEGATIVE |
|  | Neg Cont | 4 | 1.5 | 1 | 1 | 7.5 |  |  |  |  |  |  |  | N/A |
| **15** | S2-RH | 2 | 4.5 | 0.5 | 0.5 | 7.5 | 95°C x 3 min | 95°C x 10s | 52.6°C x 30s | 72°C x 45s | 72°C x 5 min | 35 | CO1 | NEGATIVE |
|  | D2-RH | 2 | 4.5 | 0.5 | 0.5 | 7.5 |  |  |  |  |  |  |  | NEGATIVE |
|  | N2-RH | 2 | 4.5 | 0.5 | 0.5 | 7.5 |  |  |  |  |  |  |  | NEGATIVE |
|  | OC2-RH | 2 | 4.5 | 0.5 | 0.5 | 7.5 |  |  |  |  |  |  |  | FAINT BAND |
|  | DC2-RH | 2 | 4.5 | 0.5 | 0.5 | 7.5 |  |  |  |  |  |  |  | NEGATIVE |
|  | Neg Cont | 2 | 4.5 | 0.5 | 0.5 | 7.5 |  |  |  |  |  |  |  | N/A |
| **16** | S2-RH | 2 | 4.5 | 0.5 | 0.5 | 7.5 | 95°C x 3 min | 95°C x 10s | 52.6°C x 30s | 72°C x 45s | 72°C x 5 min | 35 | 12S | NEGATIVE |
|  | D2-RH | 2 | 4.5 | 0.5 | 0.5 | 7.5 |  |  |  |  |  |  |  | NEGATIVE |
|  | N2-RH | 2 | 4.5 | 0.5 | 0.5 | 7.5 |  |  |  |  |  |  |  | NEGATIVE |
|  | OC2-RH | 2 | 4.5 | 0.5 | 0.5 | 7.5 |  |  |  |  |  |  |  | FAINT BAND |
|  | DC2-RH | 2 | 4.5 | 0.5 | 0.5 | 7.5 |  |  |  |  |  |  |  | NEGATIVE |
|  | Neg Cont | 2 | 4.5 | 0.5 | 0.5 | 7.5 |  |  |  |  |  |  |  | N/A |
| **17** | S2-RH | 4 | 1.5 | 1 | 1 | 7.5 | 95°C x 3 min | 95°C x 15s | 53°C x 30s | 72°C x 15s | 72°C x 5 min | 35 | CO1 | NEGATIVE |
|  | DC1-RH | 2 | 3.5 | 1 | 1 | 7.5 |  |  |  |  |  |  |  | NEGATIVE |
|  | OC2-RH | 4 | 1.5 | 1 | 1 | 7.5 |  |  |  |  |  |  |  | NEGATIVE |
|  | D3-RH | 2 | 3.5 | 1 | 1 | 7.5 |  |  |  |  |  |  |  | NEGATIVE |
|  | N3-RH | 2 | 3.5 | 1 | 1 | 7.5 |  |  |  |  |  |  |  | NEGATIVE |
|  | S1-RH | 4 | 1.5 | 1 | 1 | 7.5 |  |  |  |  |  |  |  | NEGATIVE |
|  | Neg Cont | 2 | 3.5 | 1 | 1 | 7.5 |  |  |  |  |  |  |  | N/A |
| **18** | S2-RH | 4 | 1.5 | 1 | 1 | 7.5 | 95°C x 3 min | 95°C x 15s | 53°C x 30s | 72°C x 15s | 72°C x 5 min | 35 | 12S | NEGATIVE |
|  | DC1-RH | 2 | 3.5 | 1 | 1 | 7.5 |  |  |  |  |  |  |  | NEGATIVE |
|  | OC2-RH | 4 | 1.5 | 1 | 1 | 7.5 |  |  |  |  |  |  |  | NEGATIVE |
|  | D3-RH | 2 | 3.5 | 1 | 1 | 7.5 |  |  |  |  |  |  |  | NEGATIVE |
|  | N3-RH | 2 | 3.5 | 1 | 1 | 7.5 |  |  |  |  |  |  |  | NEGATIVE |
|  | S1-RH | 4 | 1.5 | 1 | 1 | 7.5 |  |  |  |  |  |  |  | NEGATIVE |
|  | Neg Cont | 2 | 3.5 | 1 | 1 | 7.5 |  |  |  |  |  |  |  | N/A |
| **19** | S1-2-NS | 2.5 | 3 | 1 | 1 | 7.5 | 95°C x 3 min | 95°C x 15s | 53°C x 30s | 72°C x 15s | 72°C x 5 min | 35 | CO1 | NEGATIVE |
|  | S2-2-NS | 2.5 | 3 | 1 | 1 | 7.5 |  |  |  |  |  |  |  | NEGATIVE |
|  | D1-2-NS | 2.5 | 3 | 1 | 1 | 7.5 |  |  |  |  |  |  |  | NEGATIVE |
|  | D2-2-NS | 2.5 | 3 | 1 | 1 | 7.5 |  |  |  |  |  |  |  | NEGATIVE |
|  | N1-2-NS | 2.5 | 3 | 1 | 1 | 7.5 |  |  |  |  |  |  |  | NEGATIVE |
|  | OC3-2-NS | 2.5 | 3 | 1 | 1 | 7.5 |  |  |  |  |  |  |  | FAINT BAND |
|  | Neg Cont | 2.5 | 3.5 | 1 | 1 | 7.5 |  |  |  |  |  |  |  | N/A |
| **20** | S1-2-NS | 2.5 | 3 | 1 | 1 | 7.5 | 95°C x 3 min | 95°C x 15s | 53°C x 30s | 72°C x 15s | 72°C x 5 min | 35 | 12S | NEGATIVE |
|  | S2-2-NS | 2.5 | 3 | 1 | 1 | 7.5 |  |  |  |  |  |  |  | NEGATIVE |
|  | D1-2-NS | 2.5 | 3 | 1 | 1 | 7.5 |  |  |  |  |  |  |  | NEGATIVE |
|  | D2-2-NS | 2.5 | 3 | 1 | 1 | 7.5 |  |  |  |  |  |  |  | NEGATIVE |
|  | N1-2-NS | 2.5 | 3 | 1 | 1 | 7.5 |  |  |  |  |  |  |  | NEGATIVE |
|  | OC3-2-NS | 2.5 | 3 | 1 | 1 | 7.5 |  |  |  |  |  |  |  | NEGATIVE |
|  | Neg Cont | 2.5 | 3.5 | 1 | 1 | 7.5 |  |  |  |  |  |  |  | N/A |
| **21** | S1-2-NS | 2.5 | 3 | 1 | 1 | 7.5 | 95°C x 3 min | 95°C x 15s | 50°C x 30s | 72°C x 45s | 72°C x 5 min | 40 | CO1 | NEGATIVE |
|  | S2-2-NS | 2.5 | 3 | 1 | 1 | 7.5 |  |  |  |  |  |  |  | NEGATIVE |
|  | D1-2-NS | 2.5 | 3 | 1 | 1 | 7.5 |  |  |  |  |  |  |  | NEGATIVE |
|  | D2-2-NS | 2.5 | 3 | 1 | 1 | 7.5 |  |  |  |  |  |  |  | NEGATIVE |
|  | N1-2-NS | 2.5 | 3 | 1 | 1 | 7.5 |  |  |  |  |  |  |  | NEGATIVE |
|  | OC3-2-NS | 2.5 | 3 | 1 | 1 | 7.5 |  |  |  |  |  |  |  | NEGATIVE |
|  | Neg Cont | 2.5 | 3.5 | 1 | 1 | 7.5 |  |  |  |  |  |  |  | N/A |
| **22** | S1-2-NS | 2.5 | 3 | 1 | 1 | 7.5 | 95°C x 3 min | 95°C x 15s | 50°C x 30s | 72°C x 45s | 72°C x 5 min | 40 | 12S | NEGATIVE |
|  | S2-2-NS | 2.5 | 3 | 1 | 1 | 7.5 |  |  |  |  |  |  |  | NEGATIVE |
|  | D1-2-NS | 2.5 | 3 | 1 | 1 | 7.5 |  |  |  |  |  |  |  | NEGATIVE |
|  | D2-2-NS | 2.5 | 3 | 1 | 1 | 7.5 |  |  |  |  |  |  |  | NEGATIVE |
|  | N1-2-NS | 2.5 | 3 | 1 | 1 | 7.5 |  |  |  |  |  |  |  | NEGATIVE |
|  | OC3-2-NS | 2.5 | 3 | 1 | 1 | 7.5 |  |  |  |  |  |  |  | NEGATIVE |
|  | Neg Cont | 2.5 | 3.5 | 1 | 1 | 7.5 |  |  |  |  |  |  |  | N/A |
| **23** | D3-RH | 4 | 3 | 1.5 | 1.5 | 7.5 | 95°C x 3 min | 95°C x 15s | 53°C x 1 min | 72°C x 45s | 72°C x 5 min | 40 | CO1 | NEGATIVE |
|  | N3-RH | 4 | 3 | 1.5 | 1.5 | 7.5 |  |  |  |  |  |  |  | NEGATIVE |
|  | S3-RH | 4 | 3 | 1.5 | 1.5 | 7.5 |  |  |  |  |  |  |  | NEGATIVE |
|  | OS3-RH | 4 | 3 | 1.5 | 1.5 | 7.5 |  |  |  |  |  |  |  | NEGATIVE |
|  | DC3-RH | 4 | 3 | 1.5 | 1.5 | 7.5 |  |  |  |  |  |  |  | NEGATIVE |
|  | Neg Cont | 4 | 3 | 1.5 | 1.5 | 7.5 |  |  |  |  |  |  |  | NEGATIVE |
| **24** | D3-RH | 4 | 3 | 1.5 | 1.5 | 7.5 | 95°C x 3 min | 95°C x 15s | 53°C x 1 min | 72°C x 45s | 72°C x 5 min | 40 | CO1 | NEGATIVE |
|  | N3-RH | 4 | 3 | 1.5 | 1.5 | 7.5 |  |  |  |  |  |  |  | NEGATIVE |
|  | S3-RH | 4 | 3 | 1.5 | 1.5 | 7.5 |  |  |  |  |  |  |  | NEGATIVE |
|  | OS3-RH | 4 | 3 | 1.5 | 1.5 | 7.5 |  |  |  |  |  |  |  | NEGATIVE |
|  | DC3-RH | 4 | 3 | 1.5 | 1.5 | 7.5 |  |  |  |  |  |  |  | NEGATIVE |
|  | Neg Cont | 4 | 3 | 1.5 | 1.5 | 7.5 |  |  |  |  |  |  |  | NEGATIVE |
| **25** | D3-2-NS | 4 | 2.7 | 0.4 | 0.4 | 7.5 | 95°C x 3 min | 95°C x 15s | 53°C x 1 min | 72°C x 45s | 72°C x 5 min | 40 | CO1 | NEGATIVE |
|  | N3-2-RH | 4 | 2.7 | 0.4 | 0.4 | 7.5 |  |  |  |  |  |  |  | NEGATIVE |
|  | S3-2-RH | 4 | 2.7 | 0.4 | 0.4 | 7.5 |  |  |  |  |  |  |  | NEGATIVE |
|  | OS3-2-RH | 4 | 2.7 | 0.4 | 0.4 | 7.5 |  |  |  |  |  |  |  | NEGATIVE |
|  | DC3-2-RH | 4 | 2.7 | 0.4 | 0.4 | 7.5 |  |  |  |  |  |  |  | NEGATIVE |
|  | Neg Cont | 4 | 2.7 | 0.4 | 0.4 | 7.5 |  |  |  |  |  |  |  | NEGATIVE |
| **26** | D3-2-NS | 4 | 2.7 | 0.4 | 0.4 | 7.5 | 95°C x 3 min | 95°C x 15s | 53°C x 1 min | 72°C x 45s | 72°C x 5 min | 40 | 12S | NEGATIVE |
|  | N3-2-RH | 4 | 2.7 | 0.4 | 0.4 | 7.5 |  |  |  |  |  |  |  | NEGATIVE |
|  | S3-2-RH | 4 | 2.7 | 0.4 | 0.4 | 7.5 |  |  |  |  |  |  |  | NEGATIVE |
|  | OS3-2-RH | 4 | 2.7 | 0.4 | 0.4 | 7.5 |  |  |  |  |  |  |  | NEGATIVE |
|  | DC3-2-RH | 4 | 2.7 | 0.4 | 0.4 | 7.5 |  |  |  |  |  |  |  | NEGATIVE |
|  | Neg Cont | 4 | 2.7 | 0.4 | 0.4 | 7.5 |  |  |  |  |  |  |  | NEGATIVE |
| **27** | D3-2-NS | 2 | 4.7 | 0.4 | 0.4 | 7.5 | 95°C x 3 min | 95°C x 15s | 55°C x 45s | 72°C x 45s | 72°C x 5 min | 40 | CO1 | NEGATIVE |
|  | N3-2-RH | 2 | 4.7 | 0.4 | 0.4 | 7.5 |  |  |  |  |  |  |  | NEGATIVE |
|  | S3-2-RH | 2 | 4.7 | 0.4 | 0.4 | 7.5 |  |  |  |  |  |  |  | NEGATIVE |
|  | OS3-2-RH | 2 | 4.7 | 0.4 | 0.4 | 7.5 |  |  |  |  |  |  |  | NEGATIVE |
|  | DC3-2-RH | 2 | 4.7 | 0.4 | 0.4 | 7.5 |  |  |  |  |  |  |  | NEGATIVE |
|  | Neg Cont | 2 | 47 | 0.4 | 0.4 | 7.5 |  |  |  |  |  |  |  | NEGATIVE |
| **28** | D3-2-NS | 2 | 4.7 | 0.4 | 0.4 | 7.5 | 95°C x 3 min | 95°C x 15s | 55°C x 45s | 72°C x 45s | 72°C x 5 min | 40 | 12S | NEGATIVE |
|  | N3-2-RH | 2 | 4.7 | 0.4 | 0.4 | 7.5 |  |  |  |  |  |  |  | NEGATIVE |
|  | S3-2-RH | 2 | 4.7 | 0.4 | 0.4 | 7.5 |  |  |  |  |  |  |  | NEGATIVE |
|  | OS3-2-RH | 2 | 4.7 | 0.4 | 0.4 | 7.5 |  |  |  |  |  |  |  | NEGATIVE |
|  | DC3-2-RH | 2 | 4.7 | 0.4 | 0.4 | 7.5 |  |  |  |  |  |  |  | NEGATIVE |
|  | Neg Cont | 2 | 4.7 | 0.4 | 0.4 | 7.5 |  |  |  |  |  |  |  | NEGATIVE |
| **29** | N3-RH | 2 | 4.5 | 0.5 | 0.5 | 7.5 | 95°C x 3 min | 95°C x 15s | 45-55°C x 45s | 72°C x 45s | 72°C x 5 min | 35 | CO1 | 45°C - POSITIVE |
|  | N3-RH | 2 | 4.5 | 0.5 | 0.5 | 7.5 |  |  |  |  |  |  |  | 45.7°C - POSITIVE |
|  | N3-RH | 2 | 4.5 | 0.5 | 0.5 | 7.5 |  |  |  |  |  |  |  | 46.9°C - POSITIVE |
|  | N3-RH | 2 | 4.5 | 0.5 | 0.5 | 7.5 |  |  |  |  |  |  |  | 48.6°C - POSITIVE |
|  | N3-RH | 2 | 4.5 | 0.5 | 0.5 | 7.5 |  |  |  |  |  |  |  | 51°C - POSITIVE |
|  | N3-RH | 2 | 4.5 | 0.5 | 0.5 | 7.5 |  |  |  |  |  |  |  | 52.9°C - NEGATIVE |
|  | N3-RH | 2 | 4.5 | 0.5 | 0.5 | 7.5 |  |  |  |  |  |  |  | 54.2°C - NEGATIVE |
|  | Neg Cont | 2 | 4.5 | 0.5 | 0.5 | 7.5 |  |  |  |  |  |  |  | 55°C - NEGATIVE |
| **30** | N3-RH | 2 | 4.5 | 0.5 | 0.5 | 7.5 | 95°C x 3 min | 95°C x 15s | 45-55°C x 45s | 72°C x 45s | 72°C x 5 min | 35 | 12S | 45°C - POSITIVE |
|  | N3-RH | 2 | 4.5 | 0.5 | 0.5 | 7.5 |  |  |  |  |  |  |  | 45.7°C - POSITIVE |
|  | N3-RH | 2 | 4.5 | 0.5 | 0.5 | 7.5 |  |  |  |  |  |  |  | 46.9°C - POSITIVE |
|  | N3-RH | 2 | 4.5 | 0.5 | 0.5 | 7.5 |  |  |  |  |  |  |  | 48.6°C - POSITIVE |
|  | N3-RH | 2 | 4.5 | 0.5 | 0.5 | 7.5 |  |  |  |  |  |  |  | 51°C - POSITIVE |
|  | N3-RH | 2 | 4.5 | 0.5 | 0.5 | 7.5 |  |  |  |  |  |  |  | 52.9°C - POSITIVE |
|  | N3-RH | 2 | 4.5 | 0.5 | 0.5 | 7.5 |  |  |  |  |  |  |  | 54.2°C - POSITIVE |
|  | Neg Cont | 2 | 4.5 | 0.5 | 0.5 | 7.5 |  |  |  |  |  |  |  | 55°C - NEGATIVE |
| **31** | D3 | 4 | 3 | 1.5 | 1.5 | 7.5 | 95°C x 3 min | 95°C x 15s | 45°C x 45s | 72°C x 45s | 72°C x 5 min | 35 | 12S | NEGATIVE |
|  | N3 | 4 | 3 | 1.5 | 1.5 | 7.5 |  |  |  |  |  |  |  | NEGATIVE |
|  | S3 | 4 | 3 | 1.5 | 1.5 | 7.5 |  |  |  |  |  |  |  | NEGATIVE |
|  | OC3 | 4 | 3 | 1.5 | 1.5 | 7.5 |  |  |  |  |  |  |  | NEGATIVE |
|  | DC3 | 4 | 3 | 1.5 | 1.5 | 7.5 |  |  |  |  |  |  |  | NEGATIVE |
|  | Neg Cont | 4 | 3 | 1.5 | 1.5 | 7.5 |  |  |  |  |  |  |  | N/A |
| **32** | S1 | 1:10 | 5.5 | 0.5 | 0.5 | 7.5 | 95°C x 3 min | 95°C x 15s | 45°C x 45s | 72°C x 45s | 72°C x 5 min | 35 | 12S | NEGATIVE |
|  | D1 | 1:10 | 5.5 | 0.5 | 0.5 | 7.5 |  |  |  |  |  |  |  | NEGATIVE |
|  | N1 | 1:10 | 5.5 | 0.5 | 0.5 | 7.5 |  |  |  |  |  |  |  | NEGATIVE |
|  | OC1 | 1:10 | 5.5 | 0.5 | 0.5 | 7.5 |  |  |  |  |  |  |  | NEGATIVE |
|  | Neg Cont | N/A | 15 | 0 | 0 | 0 |  |  |  |  |  |  |  | N/A |
| **33** | S1 | 4 | 2.5 | 0.5 | 0.5 | 7.5 | 95°C x 3 min | 95°C x 15s | 45°C x 1 min | 72°C x 45s | 72°C x 5 min | 35 | 12S | NEGATIVE |
|  | D1 | 4 | 2.5 | 0.5 | 0.5 | 7.5 |  |  |  |  |  |  |  | FAINT BAND |
|  | N1 | 4 | 2.5 | 0.5 | 0.5 | 7.5 |  |  |  |  |  |  |  | NEGATIVE |
|  | OC1 | 4 | 2.5 | 0.5 | 0.5 | 7.5 |  |  |  |  |  |  |  | NEGATIVE |
|  | Neg Cont | 4 | 2.5 | 0.5 | 0.5 | 7.5 |  |  |  |  |  |  |  | N/A |
| **34** | S1 | 4 | 2.5 | 0.5 | 0.5 | 7.5 | 95°C x 3 min | 95°C x 15s | 45°C x 1 min | 72°C x 45s | 72°C x 5 min | 35 | CO1 | NEGATIVE |
|  | D1 | 4 | 2.5 | 0.5 | 0.5 | 7.5 |  |  |  |  |  |  |  | NEGATIVE |
|  | N1 | 4 | 2.5 | 0.5 | 0.5 | 7.5 |  |  |  |  |  |  |  | NEGATIVE |
|  | OC1 | 4 | 2.5 | 0.5 | 0.5 | 7.5 |  |  |  |  |  |  |  | NEGATIVE |
|  | Neg Cont | 4 | 2.5 | 0.5 | 0.5 | 7.5 |  |  |  |  |  |  |  | N/A |
| **35** | S1 | 1 | 8.3 | 1.5 | 1.5 | 2.7 | 94°C x 2 min | 98°C x 5s | 50°C x 10s | 72°C x 45s | 72°C x 10s | 30 | 12S | NEGATIVE |
|  | D1 | 1 | 8.3 | 1.5 | 1.5 | 2.7 |  |  |  |  |  |  |  | NEGATIVE |
|  | N1 | 1 | 8.3 | 1.5 | 1.5 | 2.7 |  |  |  |  |  |  |  | NEGATIVE |
|  | OC1 | 1 | 8.3 | 1.5 | 1.5 | 2.7 |  |  |  |  |  |  |  | NEGATIVE |
|  | Neg Cont | 1 | 8.3 | 1.5 | 1.5 | 2.7 |  |  |  |  |  |  |  | N/A |
| **36** | S1 | 4 | 2.5 | 0.5 | 0.5 | 7.5 | 94°C x 2 min | 94°C x 1 min | 48°C x 1min | 72°C x 45s | 0 | 30 | CO1 | NEGATIVE |
|  | D1 | 4 | 2.5 | 0.5 | 0.5 | 7.5 |  |  |  |  |  |  |  | NEGATIVE |
|  | N1 | 4 | 2.5 | 0.5 | 0.5 | 7.5 |  |  |  |  |  |  |  | NEGATIVE |
|  | OC1 | 4 | 2.5 | 0.5 | 0.5 | 7.5 |  |  |  |  |  |  |  | NEGATIVE |
|  | Neg Cont | 4 | 2.5 | 0.5 | 0.5 | 7.5 |  |  |  |  |  |  |  | N/A |
| **37** | S1 | 1 | 1.1 | 0.2 | 0.2 | 12.5 | 94°C x 2 min | 94°C x 1 min | 48°C x 1min | 72°C x 45s | 0 | 30 | CO1 | NEGATIVE |
|  | D1 | 1 | 1.1 | 0.2 | 0.2 | 12.5 |  |  |  |  |  |  |  | NEGATIVE |
|  | N1 | 1 | 1.1 | 0.2 | 0.2 | 12.5 |  |  |  |  |  |  |  | NEGATIVE |
|  | OC1 | 1 | 1.1 | 0.2 | 0.2 | 12.5 |  |  |  |  |  |  |  | NEGATIVE |
|  | Neg Cont | 1 | 1.1 | 0.2 | 0.2 | 12.5 |  |  |  |  |  |  |  | N/A |
| **38** | S1 | 3 | 3.5 | 0.5 | 0.5 | 7.5 | 94°C x 2 min | 94°C x 1 min | 48°C x 1min | 72°C x 45s | 0 | 40 | 12S | NEGATIVE |
|  | D1 | 3 | 3.5 | 0.5 | 0.5 | 7.5 |  |  |  |  |  |  |  | NEGATIVE |
|  | N1 | 3 | 3.5 | 0.5 | 0.5 | 7.5 |  |  |  |  |  |  |  | NEGATIVE |
|  | OC1 | 3 | 3.5 | 0.5 | 0.5 | 7.5 |  |  |  |  |  |  |  | NEGATIVE |
|  | Neg Cont | 3 | 3.5 | 0.5 | 0.5 | 7.5 |  |  |  |  |  |  |  | N/A |
| **39** | S1 | 3 | 3.5 | 0.5 | 0.5 | 7.5 | 95°C x 3 min | 95°C x 15s | 55°C x 1min | 72°C x 45s | 72°C x 5 min | 40 | CO1 | NEGATIVE |
|  | D1 | 3 | 3.5 | 0.5 | 0.5 | 7.5 |  |  |  |  |  |  |  | NEGATIVE |
|  | N1 | 3 | 3.5 | 0.5 | 0.5 | 7.5 |  |  |  |  |  |  |  | NEGATIVE |
|  | OC1 | 3 | 3.5 | 0.5 | 0.5 | 7.5 |  |  |  |  |  |  |  | NEGATIVE |
|  | Neg Cont | 3 | 3.5 | 0.5 | 0.5 | 7.5 |  |  |  |  |  |  |  | N/A |
| **40** | S1 | 2 | 4.5 | 0.5 | 0.5 | 7.5 | 95°C x 3 min | 98°C x 20s | 57°C x 10s | 72°C x 15s | 72°C x 5 min | 35 | 12S | NEGATIVE |
|  | D1 | 2 | 4.5 | 0.5 | 0.5 | 7.5 |  |  |  |  |  |  |  | NEGATIVE |
|  | N1 | 2 | 4.5 | 0.5 | 0.5 | 7.5 |  |  |  |  |  |  |  | NEGATIVE |
|  | OC1 | 2 | 4.5 | 0.5 | 0.5 | 7.5 |  |  |  |  |  |  |  | NEGATIVE |
|  | Neg Cont | 2 | 4.5 | 0.5 | 0.5 | 7.5 |  |  |  |  |  |  |  | N/A |
| **41** | S1 | 2 | 4.5 | 0.5 | 0.5 | 7.5 | 95°C x 3 min | 98°C x 20s | 57°C x 10s | 72°C x 15s | 72°C x 5 min | 35 | 12S | NEGATIVE |
|  | D1 | 2 | 4.5 | 0.5 | 0.5 | 7.5 |  |  |  |  |  |  |  | NEGATIVE |
|  | N1 | 2 | 4.5 | 0.5 | 0.5 | 7.5 |  |  |  |  |  |  |  | NEGATIVE |
|  | OC1 | 2 | 4.5 | 0.5 | 0.5 | 7.5 |  |  |  |  |  |  |  | NEGATIVE |
|  | Neg Cont | 2 | 4.5 | 0.5 | 0.5 | 7.5 |  |  |  |  |  |  |  | N/A |
| **42** | S2 | 15 | 0 | 0.5 | 0.5 | 14 | 95°C x 3 min | 98°C x 20s | 57°C x 10s | 72°C x 15s | 72°C x 5 min | 35 | 12S | NEGATIVE |
|  | SD2 | 3 | 11 | 0.5 | 0.5 | 15 |  |  |  |  |  |  |  | NEGATIVE |
|  | N2 | 10 | 4 | 0.5 | 0.5 | 15 |  |  |  |  |  |  |  | NEGATIVE |
|  | OC2 | 7 | 7 | 0.5 | 0.5 | 15 |  |  |  |  |  |  |  | NEGATIVE |
|  | Neg Cont | 7.5 | 7.5 | 0.5 | 0.5 | 15 |  |  |  |  |  |  |  | N/A |
| **43** | S2 | 4 | 2.5 | 0.5 | 0.5 | 7.5 | 95°C x 5 min | 95°C x 15s | 57°C x 1 min | 72°C x 30s | 72°C x 5 min | 40 | CO1 | NEGATIVE |
|  | D2 | 4 | 2.5 | 0.5 | 0.5 | 7.5 |  |  |  |  |  |  |  | NEGATIVE |
|  | N2 | 4 | 2.5 | 0.5 | 0.5 | 7.5 |  |  |  |  |  |  |  | NEGATIVE |
|  | OC2 | 4 | 2.5 | 0.5 | 0.5 | 7.5 |  |  |  |  |  |  |  | NEGATIVE |
|  | Neg Cont | 4 | 2.5 | 0.5 | 0.5 | 7.5 |  |  |  |  |  |  |  | N/A |
| **44** | S2 | 2 | 4.5 | 0.5 | 0.5 | 7 | 95°C x 5 min | 95°C x 15s | 57°C x 1 min | 72°C x 30s | 72°C x 5 min | 40 | CO1 | NEGATIVE |
|  | D2 | 2 | 4.5 | 0.5 | 0.5 | 7 |  |  |  |  |  |  |  | NEGATIVE |
|  | N2 | 2 | 4.5 | 0.5 | 0.5 | 7 |  |  |  |  |  |  |  | NEGATIVE |
|  | OC2 | 2 | 4.5 | 0.5 | 0.5 | 7 |  |  |  |  |  |  |  | NEGATIVE |
|  | Neg Cont | 2 | 4.5 | 0.5 | 0.5 | 7 |  |  |  |  |  |  |  | N/A |
| **45** | D1-YF | 2 | 3.5 | 1 | 1 | 7.5 | 95°C x 3 min | 95°C x 15s | 50°C x 30s | 72°C x 45s | 72°C x 7 min | 35 | CO1 | NEGATIVE |
|  | N1-YF | 2 | 3.5 | 1 | 1 | 7.5 |  |  |  |  |  |  |  | NEGATIVE |
|  | S1-YF | 2 | 3.5 | 1 | 1 | 7.5 |  |  |  |  |  |  |  | NEGATIVE |
|  | OS1-YF | 2 | 3.5 | 1 | 1 | 7.5 |  |  |  |  |  |  |  | NEGATIVE |
|  | Neg Cont | 2 | 3.5 | 1 | 1 | 7.5 |  |  |  |  |  |  |  | N/A |
| **46** | D1-YF | 1:10 | 4.5 | 1 | 1 | 7.5 | 95°C x 3 min | 95°C x 15s | 50°C x 30s | 72°C x 45s | 72°C x 7 min | 35 | CO1 | NEGATIVE |
|  | N1-YF | 1:10 | 4.5 | 1 | 1 | 7.5 |  |  |  |  |  |  |  | NEGATIVE |
|  | S1-YF | 1:10 | 4.5 | 1 | 1 | 7.5 |  |  |  |  |  |  |  | NEGATIVE |
|  | OS1-YF | 1:10 | 4.5 | 1 | 1 | 7.5 |  |  |  |  |  |  |  | NEGATIVE |
|  | Neg Cont | 1:10 | 4.5 | 1 | 1 | 7.5 |  |  |  |  |  |  |  | N/A |
| **47** | D1-YF | 2 | 5.1 | 0.3 | 0.3 | 7.5 | 94°C x 2 min | 94°C x 30s | 45°C x 1 min | 65°C x 45s | 65°C x 2 min | 35 | CO1 | NEGATIVE |
|  | N1-YF | 2 | 5.1 | 0.3 | 0.3 | 7.5 |  |  |  |  |  |  |  | NEGATIVE |
|  | S1-YF | 2 | 5.1 | 0.3 | 0.3 | 7.5 |  |  |  |  |  |  |  | NEGATIVE |
|  | OS1-YF | 2 | 5.1 | 0.3 | 0.3 | 7.5 |  |  |  |  |  |  |  | NEGATIVE |
|  | Neg Cont | 2 | 5.1 | 0.3 | 0.3 | 7.5 |  |  |  |  |  |  |  | N/A |
| **48** | D1-YF | 2 | 9.5 | 0.5 | 0.5 | 12.5 | 94°C x 3 min | 94°C x 45s | 50°C x 1 min | 72°C x 90s | 72°C x 10 min | 35 | CO1 | NEGATIVE |
|  | N1-YF | 2 | 9.5 | 0.5 | 0.5 | 12.5 |  |  |  |  |  |  |  | NEGATIVE |
|  | S1-YF | 2 | 9.5 | 0.5 | 0.5 | 12.5 |  |  |  |  |  |  |  | NEGATIVE |
|  | OS1-YF | 2 | 9.5 | 0.5 | 0.5 | 12.5 |  |  |  |  |  |  |  | NEGATIVE |
|  | Neg Cont | 2 | 9.5 | 0.5 | 0.5 | 12.5 |  |  |  |  |  |  |  | N/A |
| **49** | D3-RH | 1 | 10.5 | 0.5 | 0.5 | 12.5 | 94°C x 3 min | 94°C x 30s | 55.8°C x 1 min | 72°C x 30s | 72°C x 5min | 35 | CO1 | FAINT BAND |
|  | N3-RH | 1 | 10.5 | 0.5 | 0.5 | 12.5 |  |  |  |  |  |  |  | FAINT BAND |
|  | S3-RH | 1 | 10.5 | 0.5 | 0.5 | 12.5 |  |  |  |  |  |  |  | FAINT BAND |
|  | OS3-RH | 1 | 10.5 | 0.5 | 0.5 | 12.5 |  |  |  |  |  |  |  | NEGATIVE |
|  | Neg Cont | 1 | 10.5 | 0.5 | 0.5 | 12.5 |  |  |  |  |  |  |  | N/A |
| **50** | D1-YF | 1 | 10.5 | 0.5 | 0.5 | 12.5 | 94°C x 3 min | 94°C x 30s | 43°C x 1 min | 72°C x 30s | 72°C x 5min | 35 | CO1 | NEGATIVE |
|  | N1-YF | 1 | 10.5 | 0.5 | 0.5 | 12.5 |  |  |  |  |  |  |  | NEGATIVE |
|  | S1-YF | 1 | 10.5 | 0.5 | 0.5 | 12.5 |  |  |  |  |  |  |  | FAINT BAND |
|  | OS1-YF | 1 | 10.5 | 0.5 | 0.5 | 12.5 |  |  |  |  |  |  |  | NEGATIVE |
|  | Neg Cont | 1 | 10.5 | 0.5 | 0.5 | 12.5 |  |  |  |  |  |  |  | N/A |
| **51** | D3-NS | 1 | 10.5 | 0.5 | 0.5 | 12.5 | 94°C x 3 min | 94°C x 30s | 43°C x 1 min | 72°C x 30s | 72°C x 5min | 35 | CO1 | NEGATIVE |
|  | N3-NS | 1 | 10.5 | 0.5 | 0.5 | 12.5 |  |  |  |  |  |  |  | NEGATIVE |
|  | S3-NS | 1 | 10.5 | 0.5 | 0.5 | 12.5 |  |  |  |  |  |  |  | NEGATIVE |
|  | OS3-NS | 1 | 10.5 | 0.5 | 0.5 | 12.5 |  |  |  |  |  |  |  | NEGATIVE |
|  | Neg Cont | 1 | 10.5 | 0.5 | 0.5 | 12.5 |  |  |  |  |  |  |  | N/A |
| **52** | D3-RH | 2 | 10 | 0.4 | 0.4 | 7.2 | 95°C x 3 min | 95°C x 10s | 55.8°C x 30s | qPCR | | 40 | CO1 | UNDETERMINED |
|  | N3-RH | 2 | 10 | 0.4 | 0.4 | 7.2 |  |  |  |  |  |  |  | UNDETERMINED |
|  | S3-RH | 2 | 10 | 0.4 | 0.4 | 7.2 |  |  |  |  |  |  |  | UNDETERMINED |
|  | OS3-RH | 2 | 10 | 0.4 | 0.4 | 7.2 |  |  |  |  |  |  |  | UNDETERMINED |
|  | Neg Cont | 2 | 10 | 0.4 | 0.4 | 7.2 |  |  |  |  |  |  |  | UNDETERMINED |
| **53** | D3-NS | 2 | 10 | 0.4 | 0.4 | 7.2 | 95°C x 3 min | 95°C x 10s | 58.5°C x 30s | qPCR | | 40 | CO1 | UNDETERMINED |
|  | N3-NS | 2 | 10 | 0.4 | 0.4 | 7.2 |  |  |  |  |  |  |  | UNDETERMINED |
|  | S3-NS | 2 | 10 | 0.4 | 0.4 | 7.2 |  |  |  |  |  |  |  | UNDETERMINED |
|  | OS3-NS | 2 | 10 | 0.4 | 0.4 | 7.2 |  |  |  |  |  |  |  | UNDETERMINED |
|  | Neg Cont | 2 | 10 | 0.4 | 0.4 | 7.2 |  |  |  |  |  |  |  | UNDETERMINED |
| **54** | D1-MT | 1 | 10 | 0.4 | 0.4 | 7.2 | 95°C x 3 min | 95°C x 10s | 51.8°C x 30s | qPCR | | 40 | CO1 | UNDETERMINED |
|  | N1-MT | 1 | 10 | 0.4 | 0.4 | 7.2 |  |  |  |  |  |  |  | UNDETERMINED |
|  | S1-MT | 1 | 10 | 0.4 | 0.4 | 7.2 |  |  |  |  |  |  |  | UNDETERMINED |
|  | OS1-MT | 1 | 10 | 0.4 | 0.4 | 7.2 |  |  |  |  |  |  |  | UNDETERMINED |
|  | Neg Cont | 1 | 10 | 0.4 | 0.4 | 7.2 |  |  |  |  |  |  |  | UNDETERMINED |
| **55** | D1-NS | 1 | 10 | 0.4 | 0.4 | 7.2 | 95°C x 3 min | 95°C x 10s | 58.5°C x 30s | qPCR | | 40 | CO1 | UNDETERMINED |
|  | N1-NS | 1 | 10 | 0.4 | 0.4 | 7.2 |  |  |  |  |  |  |  | UNDETERMINED |
|  | S1-NS | 1 | 10 | 0.4 | 0.4 | 7.2 |  |  |  |  |  |  |  | UNDETERMINED |
|  | OS1-NS | 1 | 10 | 0.4 | 0.4 | 7.2 |  |  |  |  |  |  |  | UNDETERMINED |
|  | Neg Cont | 1 | 10 | 0.4 | 0.4 | 7.2 |  |  |  |  |  |  |  | UNDETERMINED |
| **56** | D1-NS | 1 | 10.5 | 0.5 | 0.5 | 12.5 | 95°C x 5 min | 95°C x 30s | 58.5°C x 30s | 72°C x 1min | 72°C x 7 min | 30 | CO1 | NEGATIVE |
|  | N1-NS | 1 | 10.5 | 0.5 | 0.5 | 12.5 |  |  |  |  |  |  |  | NEGATIVE |
|  | S1-NS | 1 | 10.5 | 0.5 | 0.5 | 12.5 |  |  |  |  |  |  |  | NEGATIVE |
|  | OS1-NS | 1 | 10.5 | 0.5 | 0.5 | 12.5 |  |  |  |  |  |  |  | NEGATIVE |
|  | Neg Cont | 1 | 10.5 | 0.5 | 0.5 | 12.5 |  |  |  |  |  |  |  | N/A |
| **57** | D1-NS | 5 | 4.2 | 0.4 | 0.4 | 10 | 95°C x 5 min | 95°C x 30s | 56.7°C x 30s | 72°C x 30s | 72°C x 7 min | 35 | CO1 | NEGATIVE |
|  | N1-NS | 5 | 4.2 | 0.4 | 0.4 | 10 |  |  |  |  |  |  |  | NEGATIVE |
|  | S1-NS | 5 | 4.2 | 0.4 | 0.4 | 10 |  |  |  |  |  |  |  | NEGATIVE |
|  | OS1-NS | 5 | 4.2 | 0.4 | 0.4 | 10 |  |  |  |  |  |  |  | NEGATIVE |
|  | Neg Cont | 5 | 4.2 | 0.4 | 0.4 | 10 |  |  |  |  |  |  |  | N/A |
| **58** | D2-NS | 5 | 4.2 | 0.4 | 0.4 | 10 | 95°C x 5 min | 95°C x 30s | 56.7°C x 30s | 72°C x 30s | 72°C x 7 min | 35 | CO1 | NEGATIVE |
|  | N2-NS | 5 | 4.2 | 0.4 | 0.4 | 10 |  |  |  |  |  |  |  | NEGATIVE |
|  | S2-NS | 5 | 4.2 | 0.4 | 0.4 | 10 |  |  |  |  |  |  |  | NEGATIVE |
|  | OS2-NS | 5 | 4.2 | 0.4 | 0.4 | 10 |  |  |  |  |  |  |  | NEGATIVE |
|  | Neg Cont | 5 | 4.2 | 0.4 | 0.4 | 10 |  |  |  |  |  |  |  | N/A |
| **59** | D1-NS | 1 | 8.2 | 0.4 | 0.4 | 10 | 95°C x 5 min | 95°C x 30s | 56.6°C x 30s | 72°C x 30s | 72°C x 7 min | 35 | CO1 | NEGATIVE |
|  | D2-NS | 1 | 8.2 | 0.4 | 0.4 | 10 |  |  |  |  |  |  |  | NEGATIVE |
|  | D3-NS | 1 | 8.2 | 0.4 | 0.4 | 10 |  |  |  |  |  |  |  | NEGATIVE |
|  | N1-NS | 1 | 8.2 | 0.4 | 0.4 | 10 |  |  |  |  |  |  |  | NEGATIVE |
|  | N2-NS | 1 | 8.2 | 0.4 | 0.4 | 10 |  |  |  |  |  |  |  | NEGATIVE |
|  | N3-NS | 1 | 8.2 | 0.4 | 0.4 | 10 |  |  |  |  |  |  |  | NEGATIVE |
|  | S1-NS | 1 | 8.2 | 0.4 | 0.4 | 10 |  |  |  |  |  |  |  | NEGATIVE |
|  | S2-NS | 1 | 8.2 | 0.4 | 0.4 | 10 |  |  |  |  |  |  |  | NEGATIVE |
|  | S3-NS | 1 | 8.2 | 0.4 | 0.4 | 10 |  |  |  |  |  |  |  | NEGATIVE |
|  | Neg Cont | 1 | 8.2 | 0.4 | 0.4 | 10 |  |  |  |  |  |  |  | N/A |
| **60** | D1-NS | 1 | 8.2 | 0.4 | 0.4 | 10 | 95°C x 3 min | 95°C x 30s | 56.6°C x 30s | 72°C x 25s | 72°C x 1 min | 35 | CO1 | NEGATIVE |
|  | D2-NS | 1 | 8.2 | 0.4 | 0.4 | 10 |  |  |  |  |  |  |  | NEGATIVE |
|  | D3-NS | 1 | 8.2 | 0.4 | 0.4 | 10 |  |  |  |  |  |  |  | NEGATIVE |
|  | N1-NS | 1 | 8.2 | 0.4 | 0.4 | 10 |  |  |  |  |  |  |  | NEGATIVE |
|  | N2-NS | 1 | 8.2 | 0.4 | 0.4 | 10 |  |  |  |  |  |  |  | NEGATIVE |
|  | N3-NS | 1 | 8.2 | 0.4 | 0.4 | 10 |  |  |  |  |  |  |  | NEGATIVE |
|  | S1-NS | 1 | 8.2 | 0.4 | 0.4 | 10 |  |  |  |  |  |  |  | NEGATIVE |
|  | S2-NS | 1 | 8.2 | 0.4 | 0.4 | 10 |  |  |  |  |  |  |  | NEGATIVE |
|  | S3-NS | 1 | 8.2 | 0.4 | 0.4 | 10 |  |  |  |  |  |  |  | NEGATIVE |
|  | Neg Cont | 1 | 8.2 | 0.4 | 0.4 | 10 |  |  |  |  |  |  |  | N/A |
| **61** | D1-YF | 5 | 4.2 | 0.4 | 0.4 | 10 | 95°C x 3 min | 95°C x 30s | 56.6°C x 30s | 72°C x 15s | 72°C x 5 min | 50 | CO1 | NEGATIVE |
|  | D2-YF | 5 | 4.2 | 0.4 | 0.4 | 10 |  |  |  |  |  |  |  | NEGATIVE |
|  | N1-YF | 5 | 4.2 | 0.4 | 0.4 | 10 |  |  |  |  |  |  |  | NEGATIVE |
|  | N2-YF | 5 | 4.2 | 0.4 | 0.4 | 10 |  |  |  |  |  |  |  | NEGATIVE |
|  | S1-YF | 5 | 4.2 | 0.4 | 0.4 | 10 |  |  |  |  |  |  |  | NEGATIVE |
|  | S2-YF | 5 | 4.2 | 0.4 | 0.4 | 10 |  |  |  |  |  |  |  | NEGATIVE |
|  | Neg Cont | 5 | 4.2 | 0.4 | 0.4 | 10 |  |  |  |  |  |  |  | N/A |
| **62** | D1-YF | 5 | 4.2 | 0.4 | 0.4 | 10 | 95°C x 3 min | 95°C x 30s | 56.6°C x 30s | 72°C x 25s | 72°C x 1 min | 40 | CO1 | NEGATIVE |
|  | D2-YF | 5 | 4.2 | 0.4 | 0.4 | 10 |  |  |  |  |  |  |  | NEGATIVE |
|  | N1-YF | 5 | 4.2 | 0.4 | 0.4 | 10 |  |  |  |  |  |  |  | NEGATIVE |
|  | N2-YF | 5 | 4.2 | 0.4 | 0.4 | 10 |  |  |  |  |  |  |  | NEGATIVE |
|  | S1-YF | 5 | 4.2 | 0.4 | 0.4 | 10 |  |  |  |  |  |  |  | NEGATIVE |
|  | S2-YF | 5 | 4.2 | 0.4 | 0.4 | 10 |  |  |  |  |  |  |  | NEGATIVE |
|  | Neg Cont | 5 | 4.2 | 0.4 | 0.4 | 10 |  |  |  |  |  |  |  | N/A |
| **63** | D1-YF | 3 | 4 | 0.5 | 0.5 | 10 | 95°C x 5 min | 95°C x 20s | 51.2°C x 15s | 72°C x 15s | 72°C x 7 min | 40 | CO1 | NEGATIVE |
|  | N2-YF | 3 | 4 | 0.5 | 0.5 | 10 |  |  |  |  |  |  |  | NEGATIVE |
|  | S3-YF | 3 | 4 | 0.5 | 0.5 | 10 |  |  |  |  |  |  |  | NEGATIVE |
|  | OS1-YF | 3 | 4 | 0.5 | 0.5 | 10 |  |  |  |  |  |  |  | NEGATIVE |
|  | Neg Cont | 3 | 4 | 0.5 | 0.5 | 10 |  |  |  |  |  |  |  | N/A |
|  | Pos Cont | 3 | 4 | 0.5 | 0.5 | 10 |  |  |  |  |  |  |  | POSITIVE |
| **64** | D1-YF | 3 | 4 | 0.5 | 0.5 | 10 | 95°C x 5 min | 95°C x 15s | 54.3°C x 30s | 72°C x 10s | 72°C x 3 min | 45 | CO1 | NEGATIVE |
|  | N2-YF | 3 | 4 | 0.5 | 0.5 | 10 |  |  |  |  |  |  |  | NEGATIVE |
|  | S3-YF | 3 | 4 | 0.5 | 0.5 | 10 |  |  |  |  |  |  |  | NEGATIVE |
|  | OS1-YF | 3 | 4 | 0.5 | 0.5 | 10 |  |  |  |  |  |  |  | NEGATIVE |
|  | Neg Cont | 3 | 4 | 0.5 | 0.5 | 10 |  |  |  |  |  |  |  | N/A |
|  | Pos Cont | 3 | 4 | 0.5 | 0.5 | 10 |  |  |  |  |  |  |  | POSITIVE |
| **65** | D1-YF | 5 | 4 | 0.5 | 0.5 | 10 | 95°C x 3 min | 95°C x 30s | 54.3°C x 30s | 72°C x 10s | 72°C x 7 min | 40 | CO1 | NEGATIVE |
|  | N2-YF | 5 | 4 | 0.5 | 0.5 | 10 |  |  |  |  |  |  |  | NEGATIVE |
|  | S1-YF | 5 | 4 | 0.5 | 0.5 | 10 |  |  |  |  |  |  |  | NEGATIVE |
|  | OS1-YF | 5 | 4 | 0.5 | 0.5 | 10 |  |  |  |  |  |  |  | NEGATIVE |
|  | Neg Cont | 5 | 4 | 0.5 | 0.5 | 10 |  |  |  |  |  |  |  | N/A |
|  | Pos Cont | 5 | 4 | 0.5 | 0.5 | 10 |  |  |  |  |  |  |  | POSITIVE |
| **66** | D1-YF | 7 | 4 | 0.5 | 0.5 | 10 | 95°C x 3 min | 95°C x 30s | 54.3°C x 30s | 72°C x 10s | 72°C x 7 min | 40 | CO1 | NEGATIVE |
|  | N2-YF | 7 | 4 | 0.5 | 0.5 | 10 |  |  |  |  |  |  |  | NEGATIVE |
|  | S1-YF | 7 | 4 | 0.5 | 0.5 | 10 |  |  |  |  |  |  |  | NEGATIVE |
|  | OS1-YF | 7 | 4 | 0.5 | 0.5 | 10 |  |  |  |  |  |  |  | NEGATIVE |
|  | Neg Cont | 7 | 4 | 0.5 | 0.5 | 10 |  |  |  |  |  |  |  | N/A |
|  | Pos Cont | 7 | 4 | 0.5 | 0.5 | 10 |  |  |  |  |  |  |  | POSITIVE |
| **67** | D3-MT | 7 | 4 | 0.5 | 0.5 | 10 | 95°C x 3 min | 95°C x 30s | 56.4°C x 30s | 72°C x 10s | 72°C x 7 min | 40 | CO1 | NEGATIVE |
|  | N3-MT | 7 | 4 | 0.5 | 0.5 | 10 |  |  |  |  |  |  |  | NEGATIVE |
|  | S3-MT | 7 | 4 | 0.5 | 0.5 | 10 |  |  |  |  |  |  |  | NEGATIVE |
|  | OS3-MT | 7 | 4 | 0.5 | 0.5 | 10 |  |  |  |  |  |  |  | NEGATIVE |
|  | Neg Cont | 7 | 4 | 0.5 | 0.5 | 10 |  |  |  |  |  |  |  | N/A |
|  | Pos Cont | 7 | 4 | 0.5 | 0.5 | 10 |  |  |  |  |  |  |  | POSITIVE |
| **68** | S1-MT | 2 | 7 | 0.5 | 0.5 | 10 | 95°C x 3 min | 95°C x 30s | 51-59°C x 30s | 72°C x 10s | 72°C x 7 min | 40 | CO1 | 51°C - FAINT BAND |
|  | S1-MT | 2 | 7 | 0.5 | 0.5 | 10 |  |  |  |  |  |  |  | 51.5°C - FAINT BAND |
|  | S1-MT | 2 | 7 | 0.5 | 0.5 | 10 |  |  |  |  |  |  |  | 52.5°C - FAINT BAND |
|  | S1-MT | 2 | 7 | 0.5 | 0.5 | 10 |  |  |  |  |  |  |  | 54.1°C - FAINT BAND |
|  | S1-MT | 2 | 7 | 0.5 | 0.5 | 10 |  |  |  |  |  |  |  | 55.9°C - FAINT BAND |
|  | S1-MT | 2 | 7 | 0.5 | 0.5 | 10 |  |  |  |  |  |  |  | 57.4°C - NEGATIVE |
|  | S1-MT | 2 | 7 | 0.5 | 0.5 | 10 |  |  |  |  |  |  |  | 58.4 °C - NEGATIVE |
|  | S1-MT | 2 | 7 | 0.5 | 0.5 | 10 |  |  |  |  |  |  |  | 59°C - NEGATIVE |
| **69** | D3-YF | 7 | 4 | 0.5 | 0.5 | 10 | 95°C x 3 min | 95°C x 30s | 59.5°C x 30s | 72°C x 10s | 72°C x 7 min | 40 | CO1 | NEGATIVE |
|  | N3-YF | 7 | 4 | 0.5 | 0.5 | 10 |  |  |  |  |  |  |  | NEGATIVE |
|  | S3-YF | 7 | 4 | 0.5 | 0.5 | 10 |  |  |  |  |  |  |  | NEGATIVE |
|  | OS3-YF | 7 | 4 | 0.5 | 0.5 | 10 |  |  |  |  |  |  |  | NEGATIVE |
|  | Neg Cont | 7 | 4 | 0.5 | 0.5 | 10 |  |  |  |  |  |  |  | N/A |
|  | Pos Cont | 7 | 4 | 0.5 | 0.5 | 10 |  |  |  |  |  |  |  | POSITIVE |
| **70** | D1-MT | 5 | 4 | 0.5 | 0.5 | 10 | 95°C x 3 min | 95°C x 30s | 56.4°C x 30s | 72°C x 15s | 72°C x 7 min | 55 | CO1 | FAINT BAND |
|  | D2-MT | 5 | 4 | 0.5 | 0.5 | 10 |  |  |  |  |  |  |  | FAINT BAND |
|  | N1-MT | 5 | 4 | 0.5 | 0.5 | 10 |  |  |  |  |  |  |  | FAINT BAND |
|  | N2-MT | 5 | 4 | 0.5 | 0.5 | 10 |  |  |  |  |  |  |  | FAINT BAND |
|  | S1-MT | 5 | 4 | 0.5 | 0.5 | 10 |  |  |  |  |  |  |  | FAINT BAND |
|  | S2-MT | 5 | 4 | 0.5 | 0.5 | 10 |  |  |  |  |  |  |  | FAINT BAND |
|  | Neg Cont | 5 | 4 | 0.5 | 0.5 | 10 |  |  |  |  |  |  |  | N/A |
| **71** | D1-YF | 5 | 4 | 0.5 | 0.5 | 10 | 95°C x 3 min | 95°C x 30s | 54.5°C x 30s | 72°C x 15s | 72°C x 7 min | 55 | CO1 | FAINT BAND |
|  | N1-YF | 5 | 4 | 0.5 | 0.5 | 10 |  |  |  |  |  |  |  | FAINT BAND |
|  | S1-YF | 5 | 4 | 0.5 | 0.5 | 10 |  |  |  |  |  |  |  | FAINT BAND |
|  | OS1-YF | 5 | 4 | 0.5 | 0.5 | 10 |  |  |  |  |  |  |  | NEGATIVE |
|  | D2-YF | 5 | 4 | 0.5 | 0.5 | 10 |  |  |  |  |  |  |  | NEGATIVE |
|  | N2-YF | 5 | 4 | 0.5 | 0.5 | 10 |  |  |  |  |  |  |  | NEGATIVE |
|  | Neg Cont | 5 | 4 | 0.5 | 0.5 | 10 |  |  |  |  |  |  |  | N/A |
| **72** | D1-NS | 10 | 4 | 0.5 | 0.5 | 10 | 95°C x 3 min | 95°C x 30s | 56.6°C x 30s | 72°C x 10s | 72°C x 7 min | 45 | CO1 | NEGATIVE |
|  | D2-NS | 10 | 4 | 0.5 | 0.5 | 10 |  |  |  |  |  |  |  | NEGATIVE |
|  | N1-NS | 10 | 4 | 0.5 | 0.5 | 10 |  |  |  |  |  |  |  | NEGATIVE |
|  | N2-NS | 10 | 4 | 0.5 | 0.5 | 10 |  |  |  |  |  |  |  | NEGATIVE |
|  | S1-NS | 10 | 4 | 0.5 | 0.5 | 10 |  |  |  |  |  |  |  | NEGATIVE |
|  | S2-NS | 10 | 4 | 0.5 | 0.5 | 10 |  |  |  |  |  |  |  | NEGATIVE |
|  | Neg Cont | 10 | 4 | 0.5 | 0.5 | 10 |  |  |  |  |  |  |  | N/A |
| **73** | D1-MT | 10 | 4 | 0.5 | 0.5 | 10 | 95°C x 3 min | 95°C x 30s | 56.6°C x 30s | 72°C x 10s | 72°C x 3 min | 45 | CO1 | NEGATIVE |
|  | N1-MT | 10 | 4 | 0.5 | 0.5 | 10 |  |  |  |  |  |  |  | NEGATIVE |
|  | S1-MT | 10 | 4 | 0.5 | 0.5 | 10 |  |  |  |  |  |  |  | NEGATIVE |
|  | OS1-MT | 10 | 4 | 0.5 | 0.5 | 10 |  |  |  |  |  |  |  | NEGATIVE |
|  | Neg Cont | 510 | 4 | 0.5 | 0.5 | 10 |  |  |  |  |  |  |  | N/A |
| **74** | D1-NS | 5 | 6.7 | 0.4 | 0.4 | 12.5 | 95°C x 3 min | 95°C x 30s | 56.6°C x 30s | 72°C x 10s | 72°C x 3 min | 45 | CO1 | NEGATIVE |
|  | N1-NS | 5 | 6.7 | 0.4 | 0.4 | 12.5 |  |  |  |  |  |  |  | NEGATIVE |
|  | S1-NS | 5 | 6.7 | 0.4 | 0.4 | 12.5 |  |  |  |  |  |  |  | NEGATIVE |
|  | OS1-NS | 5 | 6.7 | 0.4 | 0.4 | 12.5 |  |  |  |  |  |  |  | NEGATIVE |
|  | Neg Cont | 5 | 6.7 | 0.4 | 0.4 | 12.5 |  |  |  |  |  |  |  | N/A |
| **75** | D1-NS | 1 + 0.1 tissue DNA | 10.7 | 0.4 | 0.4 | 12.5 | 95°C x 3 min | 95°C x 30s | 56.6°C x 30s | 72°C x 10s | 72°C x 3 min | 45 | CO1 | NEGATIVE |
|  | N1-NS | 1 + 0.25 tissue DNA | 10.7 | 0.4 | 0.4 | 12.5 |  |  |  |  |  |  |  | POSITIVE |
|  | S1-NS | 1 + 0.50 tissue DNA | 10.7 | 0.4 | 0.4 | 12.5 |  |  |  |  |  |  |  | POSITIVE |
|  | OS1-NS | 1 + 0.75 tissue DNA | 10.7 | 0.4 | 0.4 | 12.5 |  |  |  |  |  |  |  | POSITIVE |
|  | Neg Cont | 1 | 10.7 | 0.4 | 0.4 | 12.5 |  |  |  |  |  |  |  | N/A |
|  | Pos Cont | 1 | 10.7 | 0.4 | 0.4 | 12.5 |  |  |  |  |  |  |  | POSITIVE |
